# BMP signaling is required for *nkx2*.*3*-positive pharyngeal pouch progenitor specification in zebrafish

**DOI:** 10.1371/journal.pgen.1007996

**Published:** 2019-02-14

**Authors:** Linwei Li, Guozhu Ning, Shuyan Yang, Yifang Yan, Yu Cao, Qiang Wang

**Affiliations:** 1 State Key Laboratory of Membrane Biology, Institute of Zoology, University of Chinese Academy of Sciences, Chinese Academy of Sciences, Beijing, China; 2 Institute for Stem Cell and Regeneration, Chinese Academy of Sciences, Beijing, China; Fred Hutchinson Cancer Research Center, UNITED STATES

## Abstract

Pharyngeal pouches, a series of outpocketings that bud from the foregut endoderm, are essential to the formation of craniofacial skeleton as well as several important structures like parathyroid and thymus. However, whether pharyngeal pouch progenitors exist in the developing gut tube remains unknown. Here, taking advantage of cell lineage tracing and transgenic ablation technologies, we identified a population of *nkx2*.*3*^+^ pouch progenitors in zebrafish embryos and demonstrated an essential requirement of ectodermal BMP2b for their specification. At early somite stages, *nkx2*.*3*^+^ cells located at lateral region of pharyngeal endoderm give rise to the pouch epithelium except a subpopulation expressing *pdgfαa* rather than *nkx2*.*3*. A small-scale screen of chemical inhibitors reveals that BMP signaling is necessary to specify these progenitors. Loss-of-function analyses show that BMP2b, expressed in the pharyngeal ectoderm, actives Smad effectors in endodermal cells to induce *nkx2*.*3*^+^ progenitors. Collectively, our study provides *in vivo* evidence for the existence of pouch progenitors and highlights the importance of BMP2b signaling in progenitor specification.

## Author summary

Pharyngeal pouches are essential to the formation of craniofacial skeleton as well as several important structures like parathyroid and thymus, but whether their progenitors exist in the developing gut tube remains unknown. Our study provide *in vivo* evidence that, in the early somite stages, *nkx2*.*3*^+^ cells are present in the lateral pharyngeal endoderm and give rise to the pouch epithelium. We further reveal that ectodermal BMP2b is essential for the activation of Smad effectors in endodermal cells, thereby facilitating pouch progenitor specification. Collectively, our discoveries shed new light on the cellular and molecular mechanisms of pharyngeal pouch development.

## Introduction

A prominent feature of vertebrate embryos is the presence of a series of bulges on the lateral surface of the head, which are known as pharyngeal arches. Past work has established that a key event in pharynx development is the formation of a series of outpocketings-known as pharyngeal pouches-from the foregut endoderm [1,2]. Although the number of pouches varies among vertebrates, these outpocketings are major sites expressing signaling molecules like fibroblast growth factors (FGFs) and bone morphogenetic proteins (BMPs), which have been shown to play conserved roles in establishing and patterning the pharyngeal skeleton and the induction of the arch-associated ganglia [1,3–7]. Importantly, pharyngeal pouches give rise to several important structures, including the eustachian tube, thyroid, parathyroid, thymus, and ultimobranchial body [8,9]. Pharyngeal pouch syndrome patients have underactive parathyroid glands and an underdeveloped thymus, highlighting that abnormal development of the pharyngeal pouches can result in severe human disorders [10,11]. As such, it is essential to gain a better understanding of the molecular and cellular mechanisms that occur during pharyngeal pouch development.

The segregation of the three primary germ layers-endoderm, mesoderm, and ectoderm-occurs during gastrulation. After gastrulation, a series of morphogenetic movements transforms the naive endoderm into a primitive gut tube that is surrounded by mesoderm [12]. In zebrafish, the endoderm forms two converging cell sheets by the end of gastrulation [13]. And then these cells condense at the midline to form a rod-like structure around 24 hours post-fertilization (hpf) [13,14]. During this period, the developing gut tube becomes regionalized along both the dorsal-ventral (D-V) and anterior-posterior (A-P) axes and gives rise to the progenitor cells of several digestive organs [13,14]. Dorsal pancreatic progenitors expressing high levels of *pdx1* can be detected in the most medial cells of the bilateral sheets at the 10-somite stage (14 hpf), a very early time point relative to pancreas morphogenesis [15,16]. Intestinal and ventral pancreatic progenitors expressing low levels of *pdx1* have been identified at 18 hpf in laterally located endodermal cells [17,18]. Moreover, endodermal cells expressing the liver-specific marker *ceruloplasmin* can be observed at 16 hpf, which is prior to liver bud formation, although there is no concrete evidence to demonstrate that these cells actively contribute to liver development [14,19]. Moreover, single-cell lineage tracing experiments showed that bipotential hepatopancreatic progenitors were located at least two cells away from the midline, between somites 1 and 3, at the 6–8 somite stage [20]. Taken together, these data lead to the proposal that progenitors might be specified before primary anlage formation and organ morphogenesis.

Previous data have shown that the formation of pharyngeal pouches can be divided into two phases. The initiation or segmentation stage features pharyngeal endodermal cells that are destabilized and cluster up to segmental units at discrete positions; these cells then migrate laterally in an anterior-posterior wave. The later transition stage is when these pouch-forming cells are restabilized and rearranged into epithelial bilayers [6,21]. However, whether endodermal pouch precursors exist in the developing gut tube has not been investigated in zebrafish or any other species.

Over the past few years, several signaling pathways have been suggested to be key players during pharyngeal pouch development. For example, retinoic acid signaling and FGFs expressed in head neural and mesodermal tissues are required for the segmentation and lateral migration of endodermal pouch cells [6,22,23]. Strikingly, recent work has suggested that non-canonical Wnt signaling controls distinct steps of pouch epithelial morphogenesis. To this end, Wnt11r originating from the head segmental mesoderm destabilizes the endodermal epithelium to promote the lateral movement of pouch-forming cells; comparatively, Wnt4a from the head ectoderm restabilizes the epithelium to form mature pouch bilayers. The pharynx contains at least two distinct endodermal populations: the medial and lateral pharyngeal endoderms [23]. Interestingly, inhibiting or genetically disrupting the above signaling pathways results in various defects in the morphogenesis of the lateral pouches. However, the same inhibition has no effect on the generation of medial endoderm or on the specification of the pouch endoderm [6,22,23]. These observations lead to the hypothesis that the pouch endoderm is specified during early somite stages, prior to pouch formation. However, the current lack of early specific pre-pouch markers means when, where, and how pouch progenitors originate remains unknown.

In this study, by performing a series of experiments in zebrafish embryos at the early somite stages, we identified *nkx2*.*3*^+^ endodermal cells that gave rise to the pharyngeal pouch epithelium. These pouch progenitors were located in the lateral pharyngeal endoderm, adjacent to the *nkx2*.*5*^+^ anterior lateral plate mesoderm. Using *in vivo* time-lapse image analysis, we observed that cells in the lateral-most region of the pharyneal endoderm were gradually specified into *nkx2*.*3*^+^ pouch progenitors from the 8- to 10-somite stage. Later pharmacological experiments and spatiotemporal manipulation of various signaling pathways revealed that BMP signaling was necessary but not sufficient for pouch progenitor specification. Loss-of-function analyses revealed ectodermal BMP2b was essential for the activation of Smad effectors in endodermal cells, thereby facilitating pouch progenitor specification. Unexpectively, we uncovered another subpopulation of lateral pharyngeal endoderm that was insensitive to BMP signaling and was not derived from *nkx2*.*3*^+^ pouch progenitors. Collectively, our discoveries shed new light on the cellular and molecular mechanisms of pharyngeal pouch development.

## Results

### *nkx2*.*3* marks the lateral pharyngeal endoderm at the early somite stages

**Fig 1:**
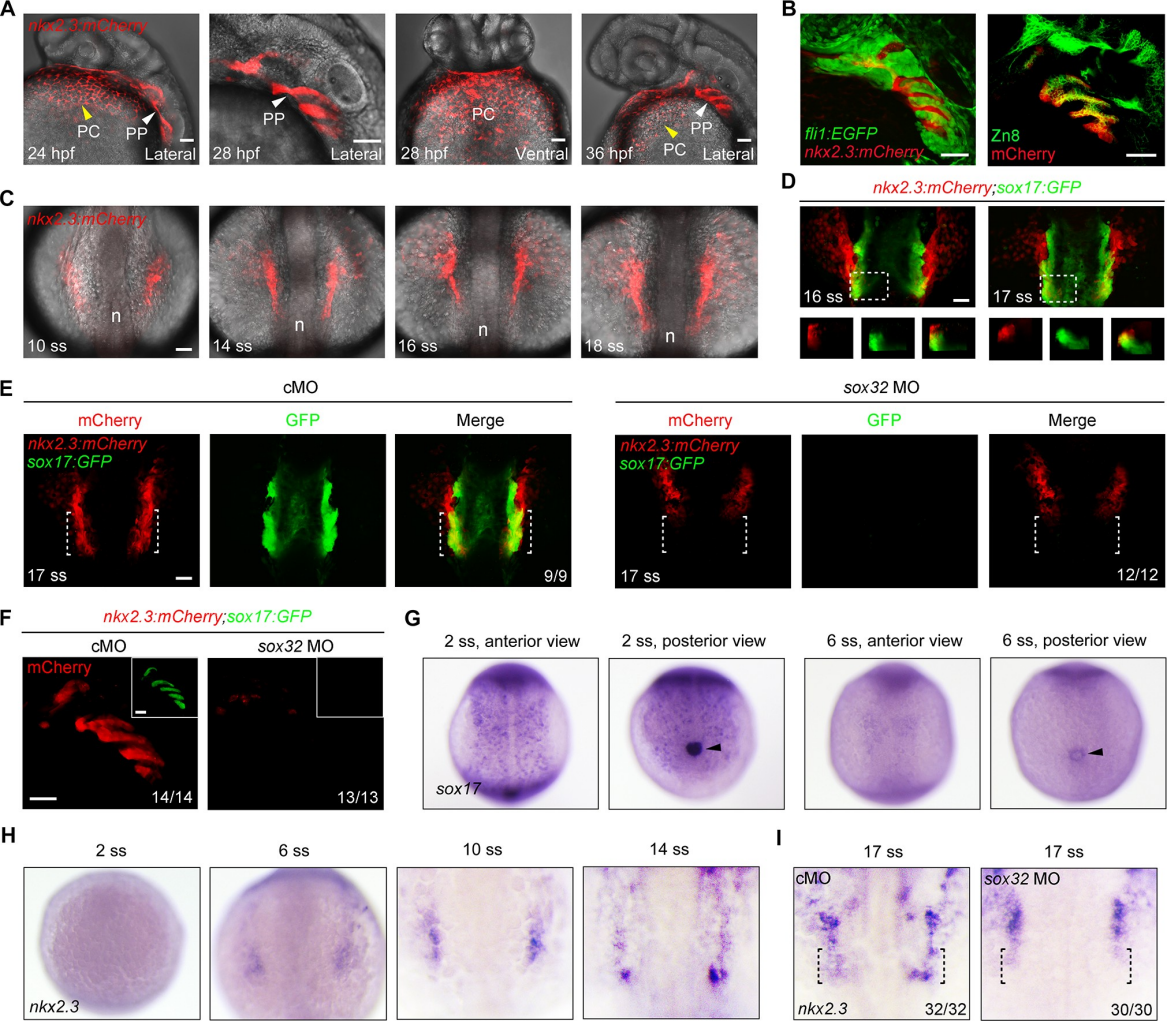
*nkx2*.*3* is expressed in the lateral pharyngeal endoderm during early somite stages. (**A**) *Tg(nkx2*.*3*:*mCherry)* embryos at 24, 28 and 36 hpf exhibiting fluorescence in the pericardium (yellow arrowhead) and pharyngeal pouches (white arrowhead). PC, pericardium; PP, pharyngeal pouch. Scale bars, 50 μm. (**B**) mCherry-positive cells (red) were located between EGFP-labeled cranial neural crest cells (green) in *Tg(nkx2*.*3*:*mCherry;fli1*:*EGFP)* embryos (left panel), and co-localized with Zn8 labeled pharyngeal pouch cells (green) in *Tg(nkx2*.*3*:*mCherry)* embryos (right panel). Scale bars, 50 μm. (**C**) mCherry fluorescence in *Tg(nkx2*.*3*:*mCherry)* embryos from the 10- to 18-somite stages. Embryos were dorsal views with anterior to the top. n, notochord; ss, somite stage. Scale bar, 50 μm. (**D**) *Tg(nkx2*.*3*:*mCherry;sox17*:*GFP)* embryos with mCherry-positive cells (red) and GFP-labeled endodermal cells (green) at the 16- and 17-somite stages. The lower panels are optical transverse sections (XZ) taken at the level of white lines in their respective upper panels. Scale bar, 50 μm. (**E-F**) mCherry (red) and GFP (green) fluorescence in *Tg(nkx2*.*3*:*mCherry;sox17*:*GFP)* embryos injected with 8 ng control MO (cMO) or 8 ng *sox32* MO at the 17-somite stage (E) and 36 hpf (F). In panel F, the GFP fluorescence was shown in the inset. The ratios of affected embryos are indicated. White dotted lines highlight the lateral pharyngeal endoderm. Scale bars, 50 μm. (**G-H**) *In situ* hybridization of *sox17* (G) and *nkx2*.*3* (H) expression in wild-type embryos at indicated developmental stages. Black arrowheads in (G) indicate the KVs. (**I**) Alteration of *nkx2*.*3* expression pattern in 8 ng *sox32* MO injected embryos at the 17-somite stage. Black dotted lines show the lateral pharyngeal endoderm.

The homeobox gene *nkx2*.*3* is robustly expressed in the endoderm epithelium lining of the pharynx from 24 hpf to 72 hpf in zebrafish embryos. As such, it is commonly used as a pharyngeal pouch marker [6,23,24]. Interestingly, whole mount *in situ* hybridization (WISH) experiments and histological section analysis indicated that the earliest *nkx2*.*3* expression occurs at the 22-somite stage (20 hpf) in bilateral, loosely arrayed clusters of pharyngeal endodermal cells [24]. To better detail *nkx2*.*3* embryonic expression *in vivo*, we generated the zebrafish transgenic line *Tg(nkx2*.*3*:*mCherry)*, in which the red fluorescent protein mCherry was expressed under control of the *nkx2*.*3* cis-regulatory sequences [21]. Analogous to the endogenous expression pattern of *nkx2*.*3*, robust mCherry fluorescence was observed in the lateral pharyngeal region by 24 hpf (Fig 1A). Soon afterwards, these mCherry-expressing cells become consolidated into paired, transverse stripes (Fig 1A). These cell stripes were located between the EGFP-labeled pharyngeal arches in *Tg(fli1*:*EGFP)* embryos and expressed the pouch epithelium adhesion protein Alcama at 36 hpf as detected by an anti-Zn8 antibody (Fig 1B). Unexpectedly, we also observed mCherry fluorescence in the pericardial region beginning at 24 hpf (Fig 1A). Co-localization studies in *Tg(nkx2*.*3*:*mCherry*;*cmlc2*:*EGFP)* embryos further confirmed that these mCherry-positive cells were located in the pericardium surrounding the heart (S1A Fig). To rule out positional effects of the transgene, *nkx2*.*3* transcript expression was examined by WISH in 36 hpf embryos. We found that *nkx2*.*3* was expressed in pericardial cells, although the pouch endoderm was overstained (S1B Fig). These results therefore suggest that the *Tg(nkx2*.*3*:*mCherry)* transgenic zebrafish can mimic the endogenous expression of *nkx2*.*3*.

We next used *Tg(nkx2*.*3*:*mCherry)* transgenic embryos to determine whether mCherry expression was detected at early somite stages. Intriguingly, time-lapse recordings from the bud (10 hpf) to 18 somite (18 hpf) stages revealed that mCherry-expressing cells first appeared in bilateral areas lying anterior to the notochord and just rostral to the future otic placodes at the 10-somite stage (14 hpf) (Fig 1C). Moreover, colocalization with *nkx2*.*5*-ZsYellow-positive cells, a bilateral populations of anterior lateral plate mesoderm that contributions to the heart and pharyngeal arch arteries [25], revealed that mCherry and ZsYellow were expressed in overlapping domains from 10- to 17-somite stages (S2A–S2C Fig). However, no cells within the paired cords were double-positive for mCherry and ZsYellow (S2A–S2C Fig), indicating that they belonged to different cell types. Interestingly, consistent with previous descriptions that the pericardium is composed of the visceral and parietal layers [26,27], we found that the mCherry^+^ cells were located in the outer layer while ZsYellow^+^ cells were housed in the inner layer in the pericardium of *Tg(nkx2*.*3*:*mCherry;nkx2*.*5*: *ZsYellow)* embryos at 28 hpf (S2D Fig).

Consistent with the expression of *nkx2*.*3*-mCherry in pericardial cells (Fig 1A and S1A Fig), fluorescence dynamics revealed that cells located in the anterior parts of the mCherry-positive fields became scattered, and then migrated anteriorly and ventrally, thereby presumably contributing to the pericardium (Fig 1C). In contrast, the posterior parts remained in the lateral pharyngeal region before the 18-somite stage (Fig 1C). Since *nkx2*.*3*-mCherry was expressed in pouches at later stages (Fig 1A), we hypothesized that the posterior regions might be the lateral pharyngeal endoderm that produces the pouches. In support of this hypothesis, we used co-localization studies in *Tg(nkx2*.*3*:*mCherry;sox17*:*GFP)* transgenic embryos and observed a subset of mCherry^+^/GFP^+^ cells in the posterior region, which were intermingled with mCherry^+^/GFP^-^ cells before and at the 16-somite stage (Fig 1D). Interestingly, most of these mCherry^+^/GFP^-^ cells migrated anteriorly by the 17-somite stage, leaving the mCherry^+^/GFP^+^ cells behind in the posterior parts (Fig 1D).

Previous work has shown that *casanova/sox32* mutant embryos lack early endoderm and all endodermal derivatives [28]. Given this, we next blocked *sox32* function by injecting antisense morpholinos (MOs) into *Tg(nkx2*.*3*:*mCherry;sox17*:*GFP)* transgenic embryos at the one-cell stage. As expected, the GFP-expressing cells completely disappeared at the 17-somite stage. Impressively, the number of mCherry-expressing cells in *sox32* MO-injected embryos was not notably changed in the anterior areas, but was dramatically reduced in the posterior areas (Fig 1E). Moreover, the formation of the endodermal pouches was completely abolished at 36 hpf in *sox32* morphants (Fig 1F). Consistent with these results, *sox32* morphants showed normal mCherry expression in the pericardium at 36 hpf (S3 Fig).

We next examined the spatiotemporal expression of *nkx2*.*3* during early somite stages by WISHs. We found that the pan-endodermal marker *sox17* was robustly expressed in the developing gut tube and the Kupffer’s vesicle (KV) at 2-somite stage (Fig 1G). Interestingly, at 6-somite stage, *sox17* expression was extremely declined in the gut endoderm, but persisted in the KV (Fig 1G). By contrast, *nkx2*.*3* transcripts first appeared at the 6-somite stage and evidently detected at the 10-somite stage in bilateral clusters located in the pharyngeal region (Fig 1H). Thus, the presence of *nkx2*.*3* transcripts follows the decline of *sox17* expression, but is 2 hours earlier than the expression of mCherry proteins in *Tg(nkx2*.*3*:*mCherry)* embryos, which is possibly due to the time requirement for protein translation. The expression of *nkx2*.*3* in the anterior parts then progressively extended anteriorly and ventrally, while remaining laterally in posterior areas at late stages (Fig 1H and 1I). Upon *sox32* MO injection, embryos only showed markedly reduced *nkx2*.*3* expression in the posterior areas at 17-somite stage (Fig 1I).

These findings reveal that, at the early somite stages, putative *nkx2*.*3*-expressing bilateral clusters contain at least two types of cells: the anterior lateral plate mesodermal cells that partly contribute to the pericardium and the lateral pharyngeal endodermal cells that likely form the pouch epithelium.

### Lineage tracing reveals the existence of pouch progenitors within the lateral pharyngeal endoderm

**Fig 2:**
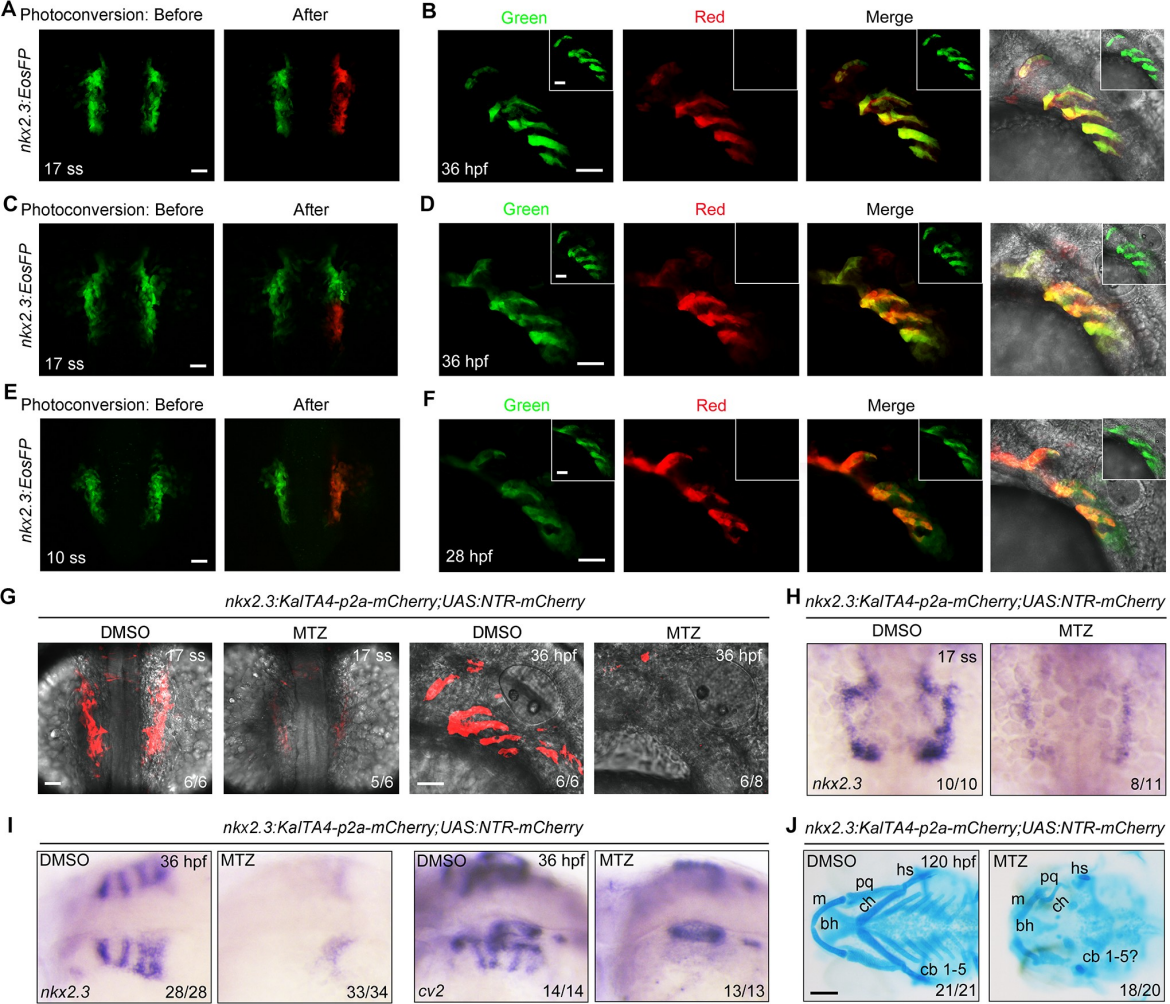
*nkx2*.*3*^+^ progenitors give rise to pharyngeal pouches. (**A-B**) *Tg(nkx2*.*3*:*EosFP)* embryos at the 17-somite stage before (green) and after (red) photoconversion in the right-side *nkx2*.*3*^+^ cluster (A). At 36 hpf, embryos were imaged in the green and red channels (B). Cells in the right-side *nkx2*.*3*^+^ cluster remained un-photoconverted as an internal control and their derivatives were imaged and shown in the inset. Scale bars, 50 μm. (**C-D**) The posterior part of the right-side *nkx2*.*3*^+^ cluster was photoconverted at the 17-somite stage (C). Images of the pharyngeal pouches in the same embryos at 36 hpf are shown in (D). Scale bars, 50 μm. (**E-F**) *Tg(nkx2*.*3*:*EosFP)* embryos were photoconverted in the right-side *nkx2*.*3*^+^ cluster at the 10-somite stage (E), and then these embryos were imaged in the red and green channels (inset) at 28 hpf (F). Scale bars, 50 μm. (**G-J**) *Tg(nkx2*.*3*:*KalTA4-p2a-mCherry;UAS*:*NTR-mCherry)* embryos were treated with 50 mM MTZ from the 32-cell stage to the 17-somite stage. Subsequently, these embryos were harvested at the indicated developmental stages for *in vivo* confocal imaging (G), *in situ* hybridization (H-I) and Alcian Blue staining (J). m, Meckel’s; pq, palatoquadrate; hs, hyosymplectic; bh, basihyal; ch, ceratohyal; cb, ceratobranchial. Scale bars, in panel G, 50 μm; in panel J, 100 μm.

Our next aim was to determine the existence of pouch-restricted progenitors in the *nkx2*.*3*^+^ lateral pharyngeal endoderm. To do this, we performed a lineage tracing analysis using Eos photoconvertible protein (EosFP), which instantly switches from green to red fluorescence following ultraviolet light exposure [29,30]. First, we photoconverted EosFPs unilaterally in the right-side *nkx2*.*3*^+^ domain in *Tg(nkx2*.*3*:*EosFP)* embryos at the 17-somite stage and kept the left-side domain as an un-photoconverted, internal control (Fig 2A). Embryos were allowed to develop to 36 hpf, at which point we observed robust red fluorescence in the right-side pharyngeal pouches. We failed to detect any red fluorescence in the contralateral side (Fig 2B). This photoconversion also resulted in many red derivatives in the right-side pericardium (S4A Fig). Consistent with the fact that a subpopulation of *nkx2*.*3*^+^ cells migrated out of the bilateral cell clusters before the photoconversion, a fraction of the right-side pericardial cells exhibited no red fluorescence (S4A Fig). Based on these observations, we conclude that *nkx2*.*3*^+^ cells in the early somite stage embryos contribute to both the developing pericardium and pouch epithelium.

To determine whether the *nkx2*.*3*^+^ cells give rise to pharyngeal pouches, we specifically photoconverted the posterior part of the right-side *nkx2*.*3*^+^ cluster at the 17-somite stage. After photoconversion, we only observed a red fluorescence signal that was specifically distributed in the relevant pouches (Fig 2C and 2D). Therefore, these findings support the conclusion that pouch epithelium is derived from *nkx2*.*3*^+^ progenitors. Furthermore, the photoconversion of *nkx2*.*3*^+^ cells at the 10-somite stage leads to red derivatives in the pouch epithelium and pericardium (Fig 2E and 2F and S4B Fig). This finding indicates the existence of the pharyngeal pouch precursors at the early somite stages, even though they are mixed with pericardial progenitors.

The above findings raised an interesting question as to whether pouch epithelium would be able to form when these *nkx2*.*3*^+^ progenitors were depleted. To answer this question, we generated a nitroreductase (NTR)-mediated ablation system [31,32], *Tg(nkx2*.*3*:*KalTA4-p2a-mCherry;UAS*:*NTR-mCherry)*, using an optimized Gal4-UAS system to drive NTR protein expression in *nkx2*.*3*^+^ cells [33]. Thus, NTR protein can catalyze the reduction of metronidazole (MTZ) into a cytotoxic product and permits the targeted ablation of the *nkx2*.*3*^+^ progenitors.

It has been suggested that NTR-mediated cell ablation can be achieved in 12–72 hours, depending on the transgenic line used [31,32]. To determine a time window of MTZ treatment for *nkx2*.*3*^+^ progenitor ablation, *Tg(nkx2*.*3*:*KalTA4-p2a-mCherry;UAS*:*NTR-mCherry)* embryos were exposed to Holtfreter’s solution with 50 mM MTZ during defined time intervals. When compared with control embryos, the embryos treated with MTZ over a time window of the 32-cell stage to the bud stage or the bud stage to the 17-somite stage exhibited unchanged mCherry expression in pouch progenitors at the 17-somite stage and in pouch epithelium at 36 hpf (S5A Fig). Similarly, the expression of pouch endoderm markers (*nkx2*.*3* and *crossveinless 2*) was not obviously changed upon such treatments (S5B Fig). However, the animals treated with MTZ from the 32-cell stage to the 17-somite stage showed markedly reduced mCherry fluorescence and *nkx2*.*3* expression at the end of the treatment (Fig 2G and 2H), indicating that most of the progenitors were successfully depleted. Furthermore, when the *nkx2*.*3*^+^ progenitors were depleted, we observed no obvious changes in pan-endoderm formation indicated by the *sox17*-GFP fluorescence in the pharyngeal region and the expression of medial pharyngeal endoderm marker *foxa1* (S5C and S5D Fig) [34], suggesting the specificity of the NTR-mediated cell ablation. These observations indicate that MTZ exposure from the 32-cell stage to the bud stage might be required for the entry of enough drugs into cells before NTR-mCherry expression. Therefore, we use the term MTZ-treated embryos to represent embryos treated with MTZ from the 32-cell stage to the 17-somite stage.

We next found that, although MTZ was washed out at the 17-somite stage, pouches remained unable to form in MTZ-treated embryos. This was indicated by the dramatically reduced expression of mCherry fluorescence and endogenous pouch markers as well as the severe loss of pharyngeal cartilage at later stages (Fig 2G and 2I and 2J). Meanwhile, MTZ-treated embryos had a large number of apoptotic pericardial cells and evident pericardial edema at 28 hpf (S5E and S5F Fig). These results further confirm the existence of pharyngeal pouch and pericardial progenitors.

### BMP signaling inhibition leads to severe loss of pharyngeal pouch progenitors

**Fig 3:**
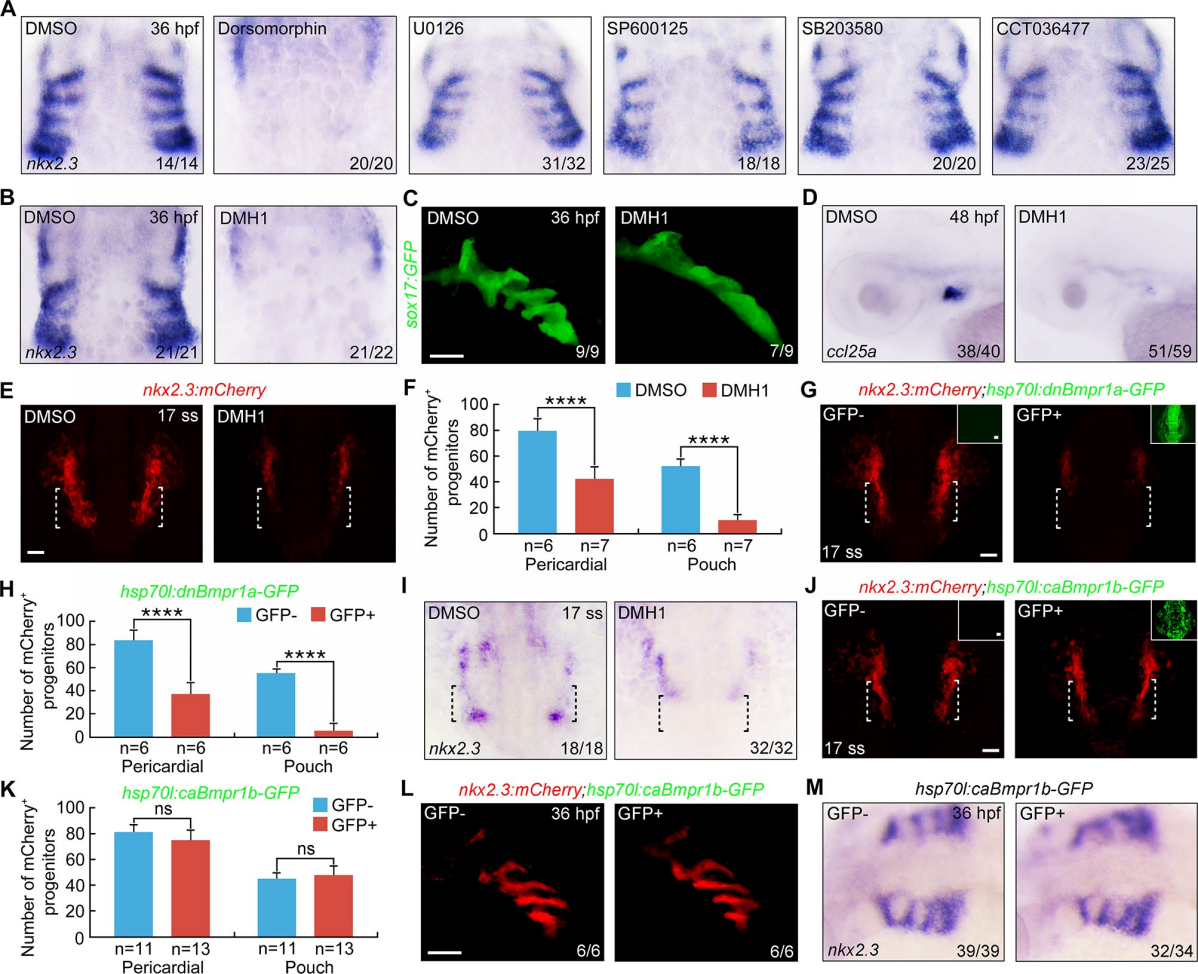
BMP signal inactivation leads to a decrease of pouch progenitors. (**A-B**) Wild-type embryos were exposed to different inhibitors from bud stage to the 17-somite stage and harvested at 36 hpf for i*n situ* hybridizations with *nkx2*.*3* probe. Dorsal views, anterior to the top. Note that embryos treated with 20 μM Dorsomorphin (A) or 10 μM DMH1 (B) showed a clear reduction of *nkx2*.*3* expression in pharyngeal pouches. (**C**) Live confocal images of malformed pharyngeal pouches in 10 μM DMH1 treated *Tg(sox17*:*GFP)* embryos at 36 hpf. Scale bar, 50 μm. (**D**) *In situ* hybridization of thymus marker *ccl25a* in DMH1 treated embryos at 48 hpf. Lateral views, anterior to the left. (**E-F**) Representative confocal sections showing mCherry^+^ progenitors in embryos treated with or without 10 μM DMH1 from bud stage to the 17-somite stage (E). The lateral pharyngeal endoderm is indicated by white dotted lines. Scale bar, 50 μm. Quantification of the numbers of pericardial and pouch progenitors positive for mCherry in DMSO and DMH1 conditions was shown in (F). The group values are expressed as mean±s.d. Student’s t-test, ****P<0.0001. (**G-H**) mCherry fluorescence in 9 hpf-heat shocked *Tg(nkx2*.*3*:*mCherry;hsp70l*:*dnBmpr1a-GFP)* embryos at the 17-somite stage (G). Scale bar, 50 μm. The numbers of mCherry+ progenitors were quantified from heat shocked-embryos with or without GFP expression (H). Student’s *t*-test, ****P<0.0001. (**I**) The expression of *nkx2*.*3* in embryos treated with 10 μM DMH1 from bud stage to the 17-somite stage. Black dotted lines indicate the region where the pouch progenitors are located. (**J-M**) Overactivation of BMP signaling has no effect on the formation of *nkx2*.*3*^+^ pouch progenitors and pouch epithelium. *Tg(nkx2*.*3*:*mCherry;hsp70l*:*caBmpr1b-GFP)* embryos were heat shocked at 9 hpf for 20 min, and then harvested at the indicated developmental stages for *in vivo* confocal imaging (J-L) and *in situ* hybridization (M). Scale bars, 50 μm. The number of mCherry^+^ progenitors was calculated from heat shocked-embryos in (J) and presented in (K). ns, non-significant.

To identify the signal pathways required for the formation of pharyngeal pouch progenitors at the early somite stages, wild-type embryos were exposed to several chemical signaling inhibitors, including Dorsomorphin (inhibitor of BMP signaling), U0126 (inhibitor of MEK1/2 signaling), SP600125 (inhibitor of JNK signaling), SB20380 (inhibitor of p38 signaling) and CCT036477 (inhibitor of Wnt signaling), from the bud to 17-somite stages. Embryos were harvested at 36 hpf and *nkx2*.*3* expression was examined using *in situ* hybridization. From this small-scale screen, we found that embryos treated with 20 μM Dorsomorphin, a selective suppressor of BMP signaling [33], exhibited significantly reduced *nkx2*.*3* expression in pharyngeal pouches (Fig 3A). We next sought to confirm that BMP signaling was required for endodermal pouch formation at early developmental stages by treating embryos in the same time window with another selective BMP pathway inhibitor (10 μM DMH1) [35,36]. DMH1 treatment led to profoundly defective pouch formation. Moreover, it also resulted in serious deficiencies in thymus anlages (as indicated by decreased *ccl25a* expression), which were derived from pharyngeal pouch epithelium (Fig 3B–3D) [37]. Collectively, these data reveal that BMP signaling is required for endodermal pouch development.

BMP signaling plays important roles in the induction and anteroposterior patterning of the endoderm during gastrulation [38,39]. However, past work has shown that blocking BMP activity at the early somite stages has no effect on early endoderm genesis [40]. To this end, DMH1-treated *Tg(sox17*:*GFP)* transgenic embryos stably expressed GFP fluorescence in unaltered expression domains at 6- and 10-somite stages when compared with control embryos, suggesting that the pan-endoderm was properly developed (S6 Fig). To investigate a possible influence of BMP signaling on pouch progenitor formation, *Tg(nkx2*.*3*:*mCherry)* transgenic embryos were treated with DMH1. Treatment started at the bud stage and lasted until the mCherry-labeled pouch progenitors were examined at the 17-somite stage. When compared with control embryos, the number of pericardial progenitors in mCherry-expressing clusters of DMH1-treated embryos was reduced (Fig 3E and 3F). However, the formation of *nkx2*.*3*^+^ pouch progenitors was nearly abolished (Fig 3E and 3F).

The decrease in pouch progenitors was also confirmed using heat-shocked *Tg(hsp70l*:*dnBmpr1a-GFP)* embryos. These embryos overexpressed the dominant negative BMP receptor 1a (dnBmpr1a) [41], excluding possible off-target effects following DMH1 treatment (Fig 3G and 3H). Consistent with previous results, DMH1-mediated BMP signal inhibition during the early somite stages also resulted in a significantly lower level of endogenous *nkx2*.*3* expression in the lateral pharyngeal endoderm at the 17-somite stage (Fig 3I). Interestingly, ectopic induction of constitutively active BMP receptor 1b (caBmpr1b) did not lead to an increase in *nkx2*.*3*^+^ pouch progenitors and pouch epithelium (Fig 3J–3M).

Together, these data suggest that BMP signaling is necessary but not sufficient for the formation of the lateral pharyngeal endoderm that contains *nkx2*.*3*^+^ pharyngeal pouch progenitors at the early somite stages.

### The lateral pharyngeal endoderm consists of BMP-sensitive and non-BMP-sensitive populations

As we had described above, the loss of *nkx2*.*3* expression and pouch structures in embryos deficient for BMP activity revealed a direct role of BMP signaling in the generation of *nkx2*.*3*^+^ pouch progenitors within the lateral pharyngeal endoderm at the early somite stages. However, a previous study showed that while BMP signaling was crucial for endodermal pouch morphogenesis, it was not necessary for lateral pharyngeal endoderm identity [40]. In this work, a lower dose (10 μM) of Dorsomorphin was used for BMP signal inhibition immediately following gastrulation, which resulted in morphological pouch defects and a reduced expression of the pouch epithelium marker *pdgfαb*, while no obvious effect on *pdgfαa* expression [40]. Given this, we speculated that BMP signaling in the lateral pharyngeal endoderm might have a dose-dependent effect.

**Fig 4:**
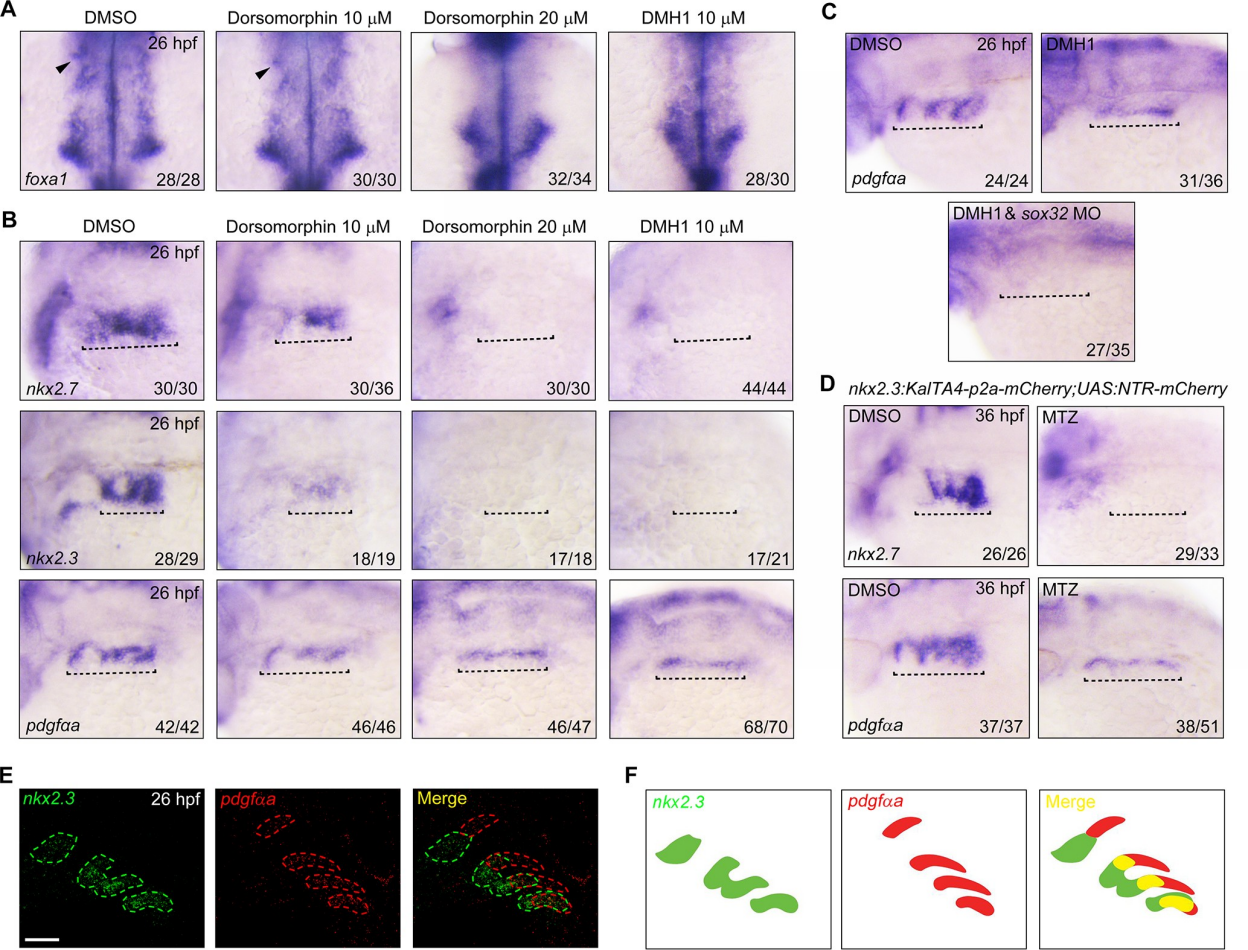
BMP signal inhibition results in a severe loss of *nkx2*.*3*^+^ pouch epithelium. (**A-B**) *In situ* hybridization analysis of the expression of *foxa1* (A), *nkx2*.*7*, *nkx2*.*3*, and *pdgfαa* (B) at 26 hpf in embryos treated with different dose of BMP inhibitors from bud stage to the 17-somite stage. Black arrowheads in (A) indicate the first pouch. Black dotted lines in (B) highlight the pharyngeal region. (C) The expression of *pdgfαa* in DMH1-treated embryos injected with or without *sox32* MO. Note that when DMH1-treated embryos were injected with 8 ng *sox32* MO, the expression of *pdgfαa* was abolished. (D) The expression of *nkx2*.*7* and *pdgfαa* in *nkx2*.*3*^+^ pouch progenitor-depleted embryos. *Tg(nkx2*.*3*:*KalTA4-p2a-mCherry;UAS*:*NTR-mCherry)* embryos were treated with 50 mM MTZ from the 32-cell stage to the 17-somite stage, and then harvested for i*n situ* hybridization at 36 hpf. (**E-F**) Double-fluorescence *in situ* hybridizations for *nkx2*.*3* and *pdgfαa* at 26 hpf, *In situ* hybridization analysis of *pdgf*α*a* at 26 hpf. Note that *nkx2*.*3* (green) and *pdgfαa* (red) display nested expression patterns along the dorsal-ventral axis of the pouch epithelium. Schematic expression of *nkx2*.*3* and *pdgfαa* was shown in (F). Scale bar, 50 μm.

To test this hypothesis, the expression of several pharyngeal endodermal markers was examined in embryos treated with different doses of Dorsomorphin from the bud to 17-somite stages. During pouch segmentation, *foxa1* is expressed not only in the medial pharyngeal endoderm but also in the first pouch [34]. Consistent with previous reports [40], the expression of *foxa1* was not notably changed in the medial pharyngeal endoderm, but obviously decreased in the first pouch at 26 hpf in embryos treated with 10 μM Dorsomorphin (Fig 4A). Meanwhile, blocking BMP signaling using 10 μM Dorsomorphin also led to decreased expression of *nkx2*.*7*, *nkx2*.*3* and *pdgfαa* in the lateral pharyngeal endoderm (Fig 4B). When compared with control animals, embryos treated with either 20 μM Dorsomorphin or 10 μM DMH1 showed no difference in the amount of medial pharyngeal endoderm (Fig 4A). However, they showed absent *foxa1*expression in the first pouch and exhibited almost no *nkx2*.*3* or *nkx2*.*7* expression in the pharyngeal region (Fig 4A and 4B). Surprisingly, *pdgfαa* expression was retained in the pharyngeal region when either DMH1 or a high Dorsomorphin dose was applied (Fig 4B). Interestingly, these *pdgfαa*^*+*^ cells were located more dorsally in treated embryos and were found in a nearly straight line, suggesting a lateral migration defect (Fig 4B). Furthermore, residual *pdgfαa*^+^ cells were completely eliminated when DMH1-treated embryos were injected with *sox32* MO (Fig 4C). This ruled out the possibility that other cell types trans-differentiated into pharyngeal endodermal cells expressing *pdgfαa* during treatment with a chemical inhibitor.

Collectively, these findings indicate that a subpopulation of lateral pharyngeal endodermal cells express *pdgfαa* but neither *nkx2*.*3* nor *nkx2*.*7*, and its development does not depend on BMP signaling. In addition, when the *nkx2*.*3*^+^ pouch progenitors in *Tg(nkx2*.*3*:*KalTA4-p2a-mCherry;UAS*:*NTR-mCherry)* embryos were ablated after early-stage MTZ treatment, the *nkx2*.*3*^+^ pouch epithelium disappeared (Fig 2G and 2I and Fig 4D). However, residual *pdgfαa*^+^ cells were restricted to the prospective dorsal region of the pouch structures (Fig 4D), further confirming the existence of an *nkx2*.*3*^*-*^/*pdgfαa*^*+*^ subpopulation within the lateral pharyngeal endoderm.

We next used other experimental approaches to investigate the cellular components within the lateral pharyngeal endoderm. Given the dorsal location of the *pdgfαa*^*+*^ cells in the BMP signal-deficient embryos, we used double fluorescence *in situ* hybridization to determine whether *nkx2*.*3* and *pdgfαa* were expressed in distinct domains. As shown in Fig 4E, in 36 hpf embryos, *nkx2*.*3*^+^/*pdgfαa*^-^ cells were enriched in the ventral-most pouch epithelium. Comparatively, *nkx2*.*3*^+^/*pdgfαa*^+^ cells were located within the intermediate domains of the endodermal pouches, while *nkx2*.*3*^-^/*pdgfαa*^+^ cells were found in the most dorsal parts of the pouch structures (Fig 4E and 4F). Thus, the lateral pharyngeal endoderm has distinct cell populations with different gene expression profiles.

When taken together, these results indicate that the lateral pharyngeal endoderm contains at least two subpopulations: one of which is *nkx2*.*3*^+^ and sensitive to BMP signaling, while the other is *nkx2*.*3*^*-*^/*pdgfαa*^*+*^ and not sensitive to BMP signaling.

### BMP signaling is required for pharyngeal pouch progenitor specification

The loss of *nkx2*.*3*^+^ cells in the lateral pharyngeal endoderm in BMP signal-deficient embryos could be due to the impaired proliferation and elevated apoptosis of pouch progenitors. Since it has been suggested that BMP signaling between 10 and 26 hpf does not play a major role in the production or survival of endodermal cells [40], we speculated that BMP signaling was required for the emergence of pouch progenitors from the lateral region of the pharyngeal endoderm. To analyze the cellular events that occurred during pouch progenitor specification, we crossed the *Tg(sox17*:*GFP)* fish to *Tg(nkx2*.*3*:*mCherry)*. Using the resulting dynamic mCherry expression, the pouch progenitor live reporter was examined by time-lapse confocal imaging.

**Fig 5:**
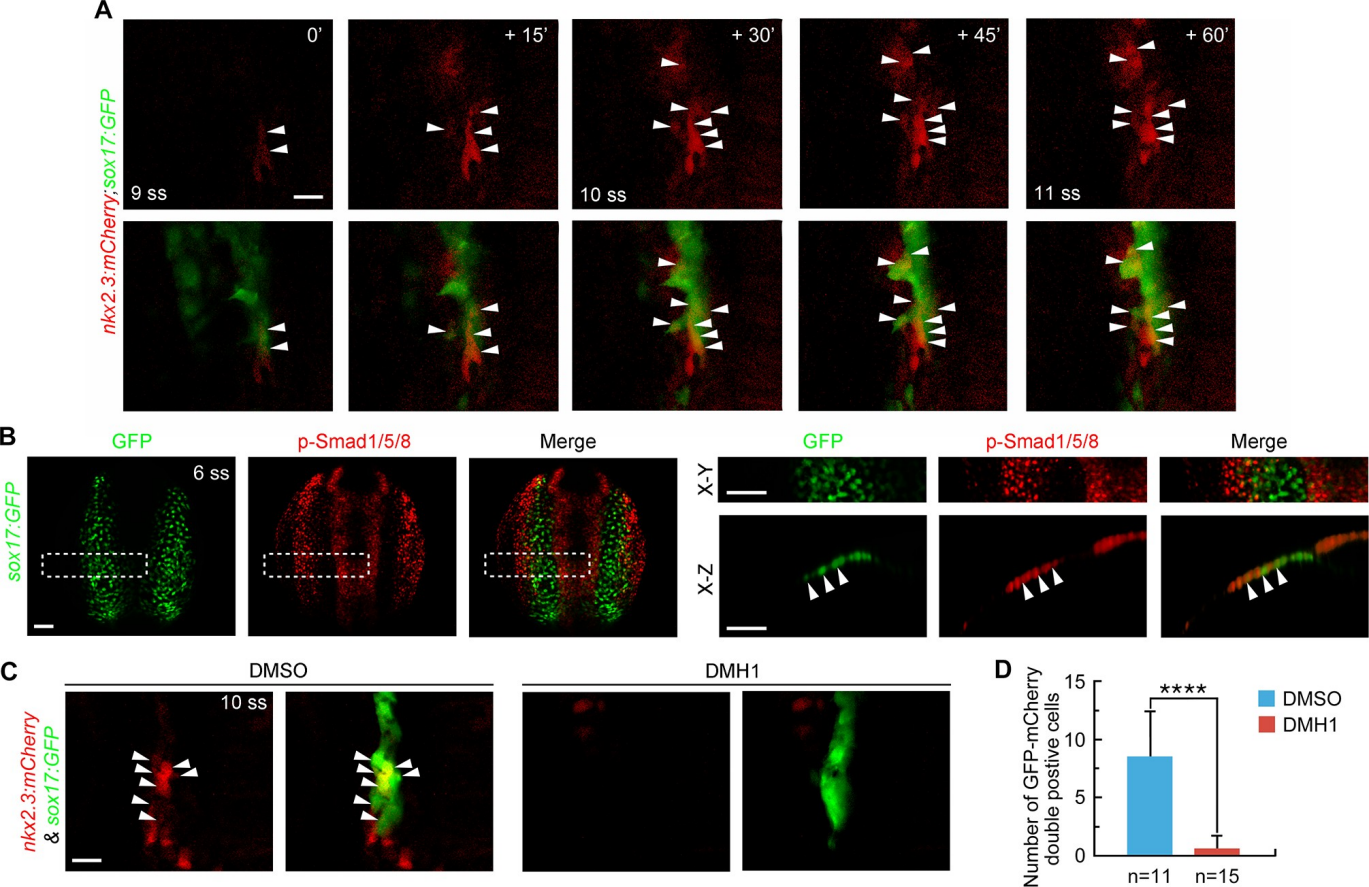
BMP signal is essential for pouch progenitor specification. (A) Time-lapse confocal images showing the specification of *nkx2*.*3*^+^ pouch progenitors from the pharyngeal endoderm. White arrowhead indicate the mCherry^+^/GFP^+^ cells in *Tg(nkx2*.*3*:*mCherry;sox17*:*GFP)* embryos. Scale bar, 20 μm. (**B**) Detection of p-Smad1/5/8 in *Tg(sox17*:*GFP)* embryos at the 6-somite stage. Representative dorsal confocal images were shown in the left three panels. Boxed areas are enlarged in the right upper panels. The orthogonal sections taken from the boxed areas were shown in the right lower panels. Arrowheads indicate the endodermal cells with p-Smad1/5/8 expression. Scale bars, 50 μm. (**C-D**) BMP signal inhibition impairs pouch progenitor specification. *Tg(nkx2*.*3*:*mCherry;sox17*:*GFP)* embryos were treated with 10 μM DMH1 from bud stage to the 10-somite stage. Representative confocal images were shown in (C). Scale bar, 20 μm. The number of mCherry^+^/GFP^+^ cells was statistical analyzed in (D). Student’s *t*-test, ****P < 0.0001.

We observed that 2 or 3 mCherry^+^/GFP^+^ cells initially appeared from the 9-somite stage (Fig 5A). Later on, more GFP^+^ cells gradually turned yellow owing to the concurrent expression of mCherry, indicating the specification of the pharyngeal endoderm cells into pouch progenitors (Fig 5A). Consistent with our prior observations that pouch progenitors emerging at the 10-somite stage could produce most of the cells in the pouch epithelium, the number of mCherry^+^/GFP^+^ cells was no longer significantly increased in transgenic embryos around the same stage (Fig 5A). This finding indicates the completion of the specification program of *nkx2*.*3*^+^ pouch progenitors.

Interestingly, previous work used the BMP response element (BRE) to drive a destabilized form of GFP (*BRE*:*d2GFP*) expression in zebrafish. This work revealed that the pharyngeal endoderm was subject to BMP signaling as early as the 10-somite stage [40]. If BMP signaling is required for the specification of pouch progenitors, it should be activated in the pharyngeal endoderm earlier than the 9-somite stage, from which the mCherry^+^/GFP^+^ cells gradually emerged. To address this issue, immunostaining was used to determine the status of phosphorylated Smad1/5/8 (p-Smad1/5/8) in *Tg(sox17*:*GFP)* embryos, which served as intracellular effectors for BMP signaling. Confocal optical sections revealed that, by the 6-somite stage (12 hpf), endogenous Smad1/5/8 was robustly activated in the lateral-most region of the pharyngeal endoderm, where pouch progenitors were presumed to be specified (Fig 5B). To determine whether reducing BMP signaling alters the emergence of pharyngeal pouch progenitors, *Tg(nkx2*.*3*:*mCherry;sox17*:*GFP)* transgenic embryos were treated with DMH1 from the bud stage. At the 10-somite stage, approximately 7 or 8 mCherry^+^/GFP^+^ cells were found in the lateral pharyngeal endoderm of control embryos. However, these cells were nearly absent in the DMH1-treated group (Fig 5C and 5D). Overall, these results suggest that BMP signaling at the early somite stages is essential for pharyngeal pouch progenitor specification.

### *bmp2b* is required for pouch formation

**Fig 6:**
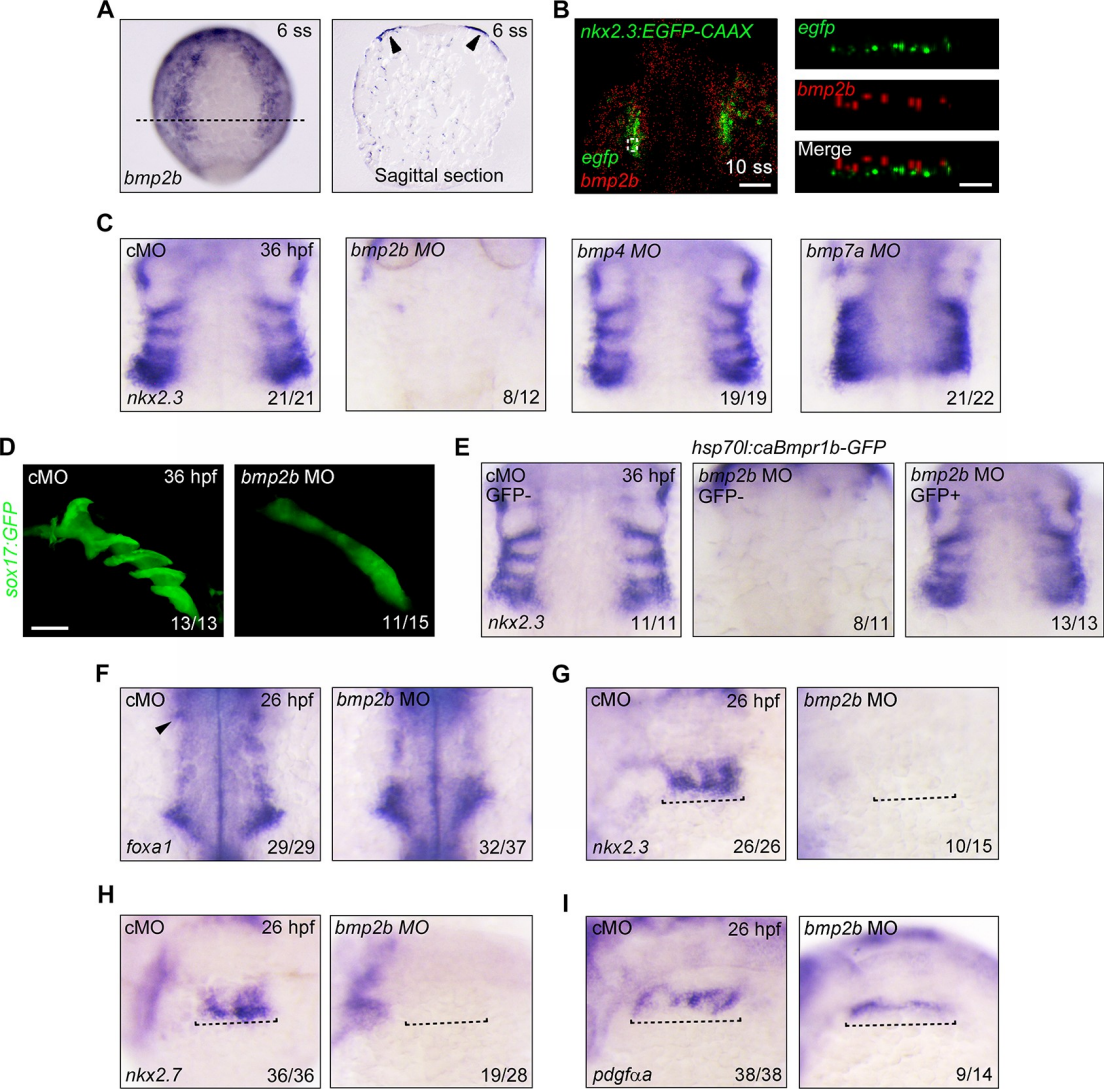
*bmp2b* is an important regulator in pouch formation. (**A**) *bmp2b* expression during early somite stages. *In situ* hybridization of *bmp2b* in whole mount embryos at the 6-somite stage (left panel). Sagittal section of the same embryo was shown in the right panel. The black dotted line indicates the plane of the section. (**B**) Whole-mount RNAscope assays showing expression of *egfp* in lateral pharyngeal endoderm (green) and *bmp2b* in pharyngeal ectoderm (red) of *Tg(nkx2*.*3*:*EGFP-CAAX)* transgenic embryos at the 10-somite stage. Left panel shows the merged sections in X-Y view (Scale bar, 50 μm); the right panel shows higher magnification orthogonal section of the boxed area in the left panel (Y-Z view; Scale bar, 10 μm). (**C**) *In situ* hybridization of *nkx2*.*3* at 36 hpf in wild-type embryos injected with 4 ng cMO, 0.25 ng *bmp2b* MO, 2 ng *bmp4* MO, and 4 ng *bmp7a* MO, respectively. Note that the expression of the pouch marker *nkx2*.*3* was not notably disrupted in *bmp4* MO or *bmp7a* MO-injected embryos, but was depleted in *bmp2b* morphants. (**D**) Malformed lateral pharyngeal endoderm (green) in 0.25 ng *bmp2b* MO-injected *Tg(sox17*:*GFP)* embryos at 36 hpf. Scale bar, 50 μm. (**E**) Overactivation of BMP signaling rescues the pouch formation in *bmp2b-*deficient embryos. *Tg(hsp70l*:*caBmpr1b-GFP)* embryos injected with 0.25 ng cMO or *bmp2b* MOs were heat shocked at 9 hpf for 20 min, and then harvested at 36 hpf for *in situ* hybridizations with *nkx2*.*3* probe. (**F-I**) The expression of *foxa1* (F), *nkx2*.*3* (G), *nkx2*.*7* (H), and *pdgfαa* (I) at 26 hpf in *bmp2b* MO-injected embryos. The black dotted lines in (G, H and G) indicate the pharyngeal regions. The black arrowheads in (F) indicate the first pouch.

Since our data suggest that BMP signaling is an important regulator of pouch progenitor specification, we next asked which BMP ligand is necessary for the formation of *nkx2*.*3*^+^ pouch epithelium. Since BMP signaling was activated in the pharyngeal endoderm at 12 hpf (Fig 5B), BMP ligands that played an essential role in determining the lateral pharyngeal endoderm identity should be expressed in the tissues adjacent to the pharyngeal endoderm at the early somite stages. It has been shown that *bmp7a* is expressed at the border of the hatching gland and in the tail bud during somitogenesis; moreover, that *bmp4* does not appear to be expressed in the pharyngeal regions until 16 hpf [40,42]. These earlier findings likely rule out the role of *bmp4* and *bmp7a* in promoting the formation of the lateral pharyngeal endoderm. In contrast, *bmp2b* expression becomes detectable in the developing pharyngeal tissues at 12 hpf and beyond [40]. In particular, transverse sections of wild-type embryos after *in situ* hybridization and co-localization analysis with *gfp* in *Tg(nkx2*.*3*:*GFP-CAAX)* embryos revealed that *bmp2b* was expressed in the pharyngeal ectoderm overlying the *nkx2*.*3*^*+*^ progenitors during the early somite stages (Fig 6A and 6B). This finding suggests that *bmp2b* might regulate the induction of lateral pharyngeal endoderm.

To investigate this issue, MOs targeting *bmp2b*, *bmp4*, and *bmp7a* were synthesized and injected individually into one-cell stage embryos. Pharyngeal pouch development was then examined at 36 hpf. As previously reported, injection of 2 ng *bmp4* MO led to left-right patterning defects, as indicated by the randomized heart jogging at 30 hpf (S7A and S7B Fig) [43]. Injection of 4 ng *bmp7a* MO resulted in strongly dorsalized phenotypes at 24 hpf (S7C and S7D Fig) [42]. However, in spite of some pouch morphological defects in *bmp4* morphants, the expression level of the pouch marker *nkx2*.*3* was not notably disrupted when either *bmp4* or *bmp7a* was depleted (Fig 6C). On the contrary, the expression of *nkx2*.*3* was absent in the pharyngeal regions of embryos injected with 0.25 ng *bmp*2b MO (Fig 6C). Further analyses showed that the pouch structures were completely missing in *Tg(sox17*:*GFP)* embryos injected with *bmp2b* MO (Fig 6D). Importantly, the defects of pouch formation in *bmp2b* morphants were evidently rescued by heat-shock-induced caBmpr1b expression (Fig 6E), suggesting BMP2b regulates the development of pouch endoderm by activation its downstream signal transduction. Altogether, these data indicate that ectodermal *bmp2b* is one of the foremost regulators in pouch formation.

Our analyses in wild-type embryos treated with BMP signal inhibitors and *Tg(nkx2*.*3*:*KalTA4-p2a-mCherry;UAS*:*NTR-mCherry)* embryos incubated with MTZ showed that the lateral pharyngeal endoderm contains both BMP-sensitive and non-sensitive subpopulations. These findings lead us to hypothesize that *bmp2b* is required only for the generation of BMP-sensitive pouch endoderm. To directly test this hypothesis, we examined the expression of multiple pharyngeal endodermal markers in *bmp*2b morphants at 26 hpf. When compared with control embryos, we found that *bmp*2b morphants had normal development of the medial pharyngeal endoderm with *foxa1* expression (Fig 6F). However, the expression of *foxa1* in the first pouch and the expression of *nkx2*.*3* and *nkx2*.*7* in the lateral pharyngeal endoderm were absent (Fig 6F, 6G and 6H). Strikingly, at this stage, the BMP non-sensitive pouch endoderm that was marked by *pdgfαa* expression could be easily detected in *bmp2b-*deficient embryos without the presence of lateral distributions (Fig 6I).

Collectively, these data demonstrate that *bmp2b* signaling mediates the genesis of BMP-sensitive lateral pharyngeal endoderm during pouch formation.

### *bmp2b* is indispensable for pharyngeal pouch progenitor specification

**Fig 7:**
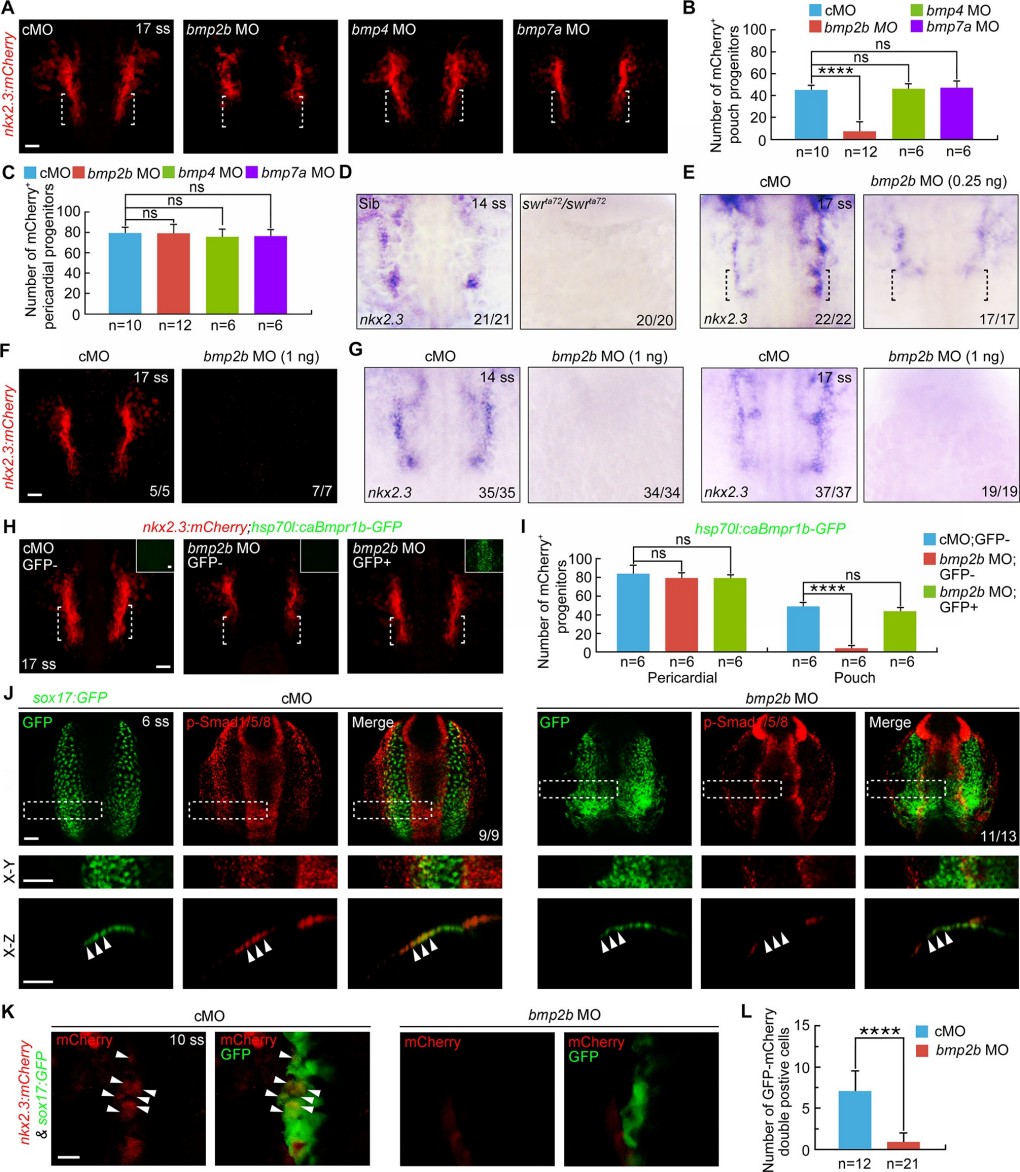
*bmp2b* is necessary for pharyngeal pouch progenitor specification. (**A**) mCherry fluorescence at the 17-somite stage in *Tg(nkx2*.*3*:*mCherry)* embryos injected with 4 ng cMO, 0.25 ng *bmp2b* MO, 2 ng *bmp4* MO, and 4 ng *bmp7a* MO, respectively. Scale bar, 50 μm. (**B-C**) Quantification of the number of the mCherry^+^ pouch progenitors (B) and the pericardial progenitors (C) from embryos of each group shown in (A). Student’s *t*-test, ****P<0.0001. ns, non-significant. (**D-E**) Alteration of the *nkx2*.*3* expression pattern in *swr*^*ta72*^ mutants at the 14-somite stage (D) and embryos injected with 0.25 ng *bmp2b* MO at the 17-somite stage (E). Black dotted lines indicate the region where the pouch progenitors are located. (**F**) mCherry fluorescence at the 17-somite stage in *Tg(nkx2*.*3*:*mCherry)* embryos injected with 1 ng cMO or *bmp2b* MO. Scale bar, 50 μm. (**G**) The expression of *nkx2*.*3* in wild-type embryos injected with indicated MOs. (**H-I**) mCherry fluorescence in heated *Tg(nkx2*.*3*:*mCherry;hsp70l*:*caBmpr1b-GFP)* embryos. Embryos injected with 0.25 ng cMO or *bmp2b* MOs were heat shocked at 9 hpf for 20 min, and then harvested at the 17-somite stage for confocal imaging (H). Scale bar, 50 μm. The numbers of mCherry^+^ pericardial and pouch progenitors were quantified from heat shocked-embryos with or without GFP expression (I). The group values are expressed as mean±s.d. Student’s t-test, ****P<0.0001. ns, non-significant. (**J**) p-Smad1/5/8 levels were dramatically reduced in in the pharyngeal endoderm of *bmp2b* morphants. cMO or *bmp2b* MO-injected *Tg(sox17*:*GFP)* embryos at the 6-somite stage were stained for anti-p-smad1/5/8 and anti-GFP antibodies. Representative dorsal confocal images were shown in the upper panels. Boxed areas are enlarged and presented in the middle panels. The orthogonal sections taken from the boxed areas were shown in the lower panels. Scale bar, 50 μm. (**K-L**) Knocking down *bmp2b* resulted in the failure of pharyngeal pouch progenitor specification. *Tg(nkx2*.*3*:*mCherry;sox17*:*GFP)* embryos were injected with ng *bmp2b* MO at one-cell stage and harvested at the 10-somite stage for confocal imaging analysis (K). Arrowheads indicate the pouch progenitors expression both mCherry and GFP. Scale bar, 20 μm. The number of mCherry^+^/GFP^+^ cells was statistical analyzed in (L). Student’s *t*-test, ****P < 0.0001.

We next performed knockdown experiments in the *Tg(nkx2*.*3*:*mCherry)* transgenic line to explore whether the defective development of pharyngeal pouches in *bmp2b-*deficient embryos was due to the loss of pouch progenitors. Consistent with the defects of pouch formation in *bmp2b* morphants (Fig 6C), MOs targeting *bmp2b* but not *bmp4* or *bmp7a* significantly reduced the number of pouch progenitors (Fig 7A and 7B). However, the pericardial progenitors appeared to be normally induced at the 17-somite stage (Fig 7A and 7C). To confirm these results, we examined endogenous *nkx2*.*3* expression at the 14-somite stage in *swr*^*ta72*^ mutants carrying a mutated *bmp2b* gene, which encodes inactive BMP2b mutant proteins with additional dominant negative functions [44]. This period was chosen since after that, they began to die [39,44]. As descripted in previous findings [39], although the convergence of *sox17-*expressing endodermal cells was slightly delayed in *swr*^*ta72*^ mutants at bud stage, the endoderm specification was apparently unaffected (S8 Fig). To our surprise, *nkx2*.*3* transcripts were completely absent in *swr*^*ta72*^ mutant embryos (Fig 7D), indicating the absence of both pharyngeal pouch and pericardial progenitors. These observations combined with the finding that *bmp2b* is expressed in the developing pharyngeal tissues during early somite stages, imply that the absence of pouch progenitors in *swr*^*ta72*^ mutants is due to the loss of *bmp2b* activity instead of the dominant negative effects of BMP2b mutant proteins that interfere with other BMPs in the embryos.

The differential presence of the pericardial progenitors in the mutants and morphants may represent different losses of *bmp2b* activity. To support this hypothesis, we found that embryos injected with 0.25 ng *bmp2b* MO showed a distinct decrease in *nkx2*.*3* expression in regions where only the pouch progenitors while not the pericardial progenitors were located at the 17-somite stage (Fig 7E). However, injection of 1 ng *bmp2b* MO into *Tg(nkx2*.*3*:*mCherry)* or wild-type embryos resulted in the near absence of the pouch and pericardial progenitors, a phenotype similar to that of *swr*^*ta72*^ mutants (Fig 7F and 7G). In addition, we found a significant recovery of the decrease in pouch progenitor number induced by *bmp2b*-depletion in heat-shocked *Tg(hsp70l*:*caBmpr1b-GFP)* embryos (Fig 7H and 7I). These results indicate that BMP signaling triggered by BMP2b plays a central role in the specification of pouch progenitors.

Finally, we used immunostaining with anti-p-Smad1/5/8 antibody to examine the activation of BMP signal in the pharyngeal endoderm of *bmp2b* MO-injected *Tg(sox17*:*GFP)* embryos. When compared with control embryos, the p-Smad1/5/8 level was decreased in the ventral-most pharyngeal endodermal cells in *bmp2b* morphants (Fig 7J). The insufficient BMP signal activity in pharyngeal endoderm would likely impair pouch progenitor specification. To this end and similar to the effects of DMH1-treatment, *bmp2b* MO injection severely impeded the specification of *nkx2*.*3*^+^ pouch progenitors at the 10-somite stage (Fig 7K and 7L).

Together, these data suggest that the ectoderm expressing *bmp2b* enables the adjacent pharyngeal endoderm to receive sufficient BMP signal (S9 Fig). In this way, the differentiation of endodermal cells into pouch progenitors is promoted during the early somite stages.

## Discussion

The pharyngeal pouches play essential roles in patterning the surrounding arches. Moreover, they play an important role in the development of a variety of essential structures like the ear drum and glands such as the thymus and parathyroid [9]. Developmental defects of these pouches lead to a number of human hereditary disorders, such as DiGeorge and Branchio-Oto-Renal syndromes [9–11]. Thus, a better understanding of how they develop from the foregut is key to a better mechanistic view of such human disorders. However, although pouch morphogenesis and the formation of pouch derivatives have been described in different animals [9], the existence of pharyngeal pouch progenitors in the developing endoderm has remained an open question. Our study provides *in vivo* evidence that, in the early somite stages, *nkx2*.*3*^+^ cells are present in the lateral pharyngeal endoderm and give rise to the pouch epithelium. *In vivo* time-lapse image analysis revealed that these pouch progenitors were gradually specified from endodermal cells from the 9- to 10-somite stage. Furthermore, our data indicated that *bmp2b*, which is expressed in the pharyngeal ectoderm from the 6-somite stage, triggered robust BMP activity in endodermal cells. This signaling thus promotes the emergence of pouch progenitors.

An unanticipated finding is that a non-endodermal population of *nkx2*.*3*^+^ cells are mingled with pouch progenitors when they are initially specified. These cells then reside in the anterior parts of the *nkx2*.*3*-positive fields after cell migration, ultimately comprising progenitor cells for the pericardium. Interestingly, our cell-lineage tracing analysis revealed that the pericardial progenitors located on one side of the *nkx2*.*3*-positive bilateral clusters were only able to form the ipsilateral pericardium. Past work regarding this issue has been somewhat consistent with our observations. More specifically, studies have shown that while the etiology of congenital pericardial defects is poorly understood, it can be characterized by the location of the defects (e.g., left pericardial absence, right pericardial absence and bilateral pericardial absence) [26,27]. Congenital defects of the pericardium are generally considered to be a consequence of failure of the pleuropericardial membrane to close during development [26,45]. In fact, when the *nkx2*.*3*^+^ pericardial progenitors were depleted at the early somite stages in zebrafish embryos using the MTZ/NTR system, severe pericardial edema was observed at later stages. Given these findings, it is possible that pericardial deficiencies might also result from the failure of pericardial progenitors to generate on either one or both sides.

In the somite-stage *Tg(nkx2*.*3*:*mCherry;sox17*:*GFP)* transgenic embryos, pharyngeal pouch progenitors expressed both mCherry and GFP, while pericardial progenitors only expressed mCherry. Although these two different progenitor types were distinguished by their different locations after developmentally-regulated cell migration, the future identification of endogenous biomolecular markers for each type of progenitors will aid the study of cell fate determination.

During the early somite stages of vertebrate development, signals from surrounding tissues induce the specification of lineage-specific progenitors for several foregut organs [12]. For example, a study using mouse embryos showed that the development of dorsal pancreatic precursors required retinoic acid signaling that originated from the adjacent lateral plate mesoderm [46]. In zebrafish, *wnt2bb* is expressed in restricted, bilateral domains of the lateral plate mesoderm and directly induces the adjacent endoderm to form liver anlage [47]. Single-cell-labeling experiments in zebrafish has provided *in vivo* evidence that the liver and pancreas originate from common progenitors; moreover, that proximity to the source of mesodermal *bmp2b* influences cell fate [20]. Previous data have also suggested that, during pharyngeal pouch morphogenesis, Wnt signals from the mesoderm and ectoderm mediate the initial destabilization and subsequent remodeling of the pouch epithelium [21]. However, the actual identity and origin of these signals that determine the fate of pharyngeal pouch progenitors remains unknown.

Using results from a small-scale screen of chemical inhibitors, we identified BMP signaling was required to specify pouch progenitors from the head endoderm during the early somite stages. Subsequent to this finding, we determined that the BMP ligand gene *bmp2b*-but not *bmp4* or *bmp7a-*played an essential role in pouch progenitor specification. *bmp2b* is dynamically expressed in multiple tissues during the gastrulation and segmentation stages and has been shown to be important for multiple stages of endoderm development [20,39]. Although we were unable to inactivate *bmp2b* expression in a spatio-temporal manner during zebrafish embryo development, several independent lines of evidence converge in support of pharyngeal ectoderm-derived BMP2b as an important regulator of the induction of *nkx2*.*3*^+^ pouch progenitors. More specifically: (1) Endogenous Smad1/5/8 proteins, the intracellular effectors of BMP signaling, are activated in the lateral-most region of the head endoderm as early as the 6-somite stage. (2) Inhibition of BMP signaling during the early somite stages by either applying BMP inhibitors or overexpressing a dominant-negative BMP receptor significantly decrease the number of pouch progenitors. This suggests that BMP signaling is required for cell fate determination. (3) *bmp2b* begins to be expressed from the 6-somite stage in the pharyngeal ectoderm overlying the *nkx2*.*3*^+^ pouch progenitors. (4) Inactivation of *bmp2b* using an antisense morpholino does not notably impact the formation of the medial pharyngeal endoderm, but does impair the phosphorylation of Smad1/5/8 and the specification of pouch progenitors. Interestingly, ectopic induction of BMP signaling cannot induce more pouch progenitors during the early somite stages and cannot increase pouch epithelium at later stages. Collectively, these findings imply that BMP signaling is necessary but not sufficient for pouch progenitor specification.

Our results unambiguously demonstrate the existence of *nkx2*.*3*^+^ pouch progenitors in the lateral pharyngeal endoderm. Disrupting BMP signaling through the application of chemical inhibitors such as Dorsomorphin and DMH1 during early somite stages disturbs the generation of the lateral pharyngeal endoderm. Ultimately, this leads to severe loss of the pouch epithelium. These observations are different from previous findings, which indicated that BMP signaling was necessary for proper pouch morphogenesis, but not crucial to the genesis of the pouch epithelium [40]. This discrepancy could simply be due to the lower dose (10 μM) of Dorsomorphin used by Lovely and colleagues, which may have resulted in a negligible decrease in expression of the pouch epithelium marker *pdgfαa* [40]. However, when we treated embryos with a higher dose (20 μM) of Dorsomorphin or 10 μM DMH1, a more efficient BMP signal inhibitor, we found that *nkx2*.*3* and *nkx2*.*7* expression were almost completely abolished in the pharyngeal region. Comparatively, *pdgfαa* expression in the pharyngeal region was retained. Based on these observations, we speculate that, excepting the *nkx2*.*3*^+^ cells, the lateral pharyngeal endoderm might contain another distinct cell population expressing *pdgfαa*, but not *nkx2*.*3*. Moreover, its development dose not dependent on BMP signaling. In support of this hypothesis, our studies of MTZ-induced depletion of *nkx2*.*3*^+^ pouch progenitors showed that the *nkx2*.*3*^+^ pouch epithelium was disrupted, while the *nkx2*.*3*^*-*^/*pdgfaa*^*+*^ lateral pharyngeal endoderm persisted. This finding also suggests that the *nkx2*.*3*^*-*^/*pdgfaa*^*+*^ pouch epithelium should not be derived from *nkx2*.*3*^+^ pouch progenitors. In the future, it will be interesting to discern whether a subset of pouch progenitors exist and contribute to *nkx2*.*3*^*-*^/*pdgfaa*^*+*^ lateral pharyngeal endoderm.

Another interesting observation is that *nkx2*.*3* and *pdgfαa* display nested expression patterns along the dorsal-ventral (DV) axis of the pouch epithelium. In particular, *nkx2*.*3*^+^ cells are located in the ventral regions of each pouch. When *nkx2*.*3*^+^ pouch epithelium development was eliminated, it seemed that the residual *pdgfαa*^+^ cells were incapable of migrating laterally. This was evidenced by their nearly straight-line, dorsal location. Time-lapse microscopy studies revealed a collective cell migration during the pouch epithelium outgrowth [21]. It is therefore possible that the *nkx2*.*3*^+^ pouch epithelium functions as the leading edge of cells that direct the migratory behavior for the collective cell migration [48]. Similar regional expression of distinct genes has been well described in pharyngeal arch neural crest cells [49–51]. Previous studies have showed that these DV domains in each arch are induced by signals from the surrounding epithelia, including the endoderm and ectoderm [7,52]. The establishment of the DV patterning in developing arches is an important process for the fate determination of neural crest cells during craniofacial skeleton development [49,53,54]. Thus, there is a clear need for further, in-depth studies to uncover the mechanism for the DV patterning of pouch epithelium; moreover, to disclose the developmental function of pouch epithelial regionalization.

## Materials and methods

### Ethics statement

Our zebrafish experiments were all approved and carried out in accordance with the Animal Care Committee at the Institute of Zoology, Chinese Academy of Sciences (Permission Number: IOZ-13048).

### Zebrafish strains

Zebrafish strains were maintained in stand laboratory conditions. Embryos were obtained from natural zebrafish matings, raised in Holtfreter’s solution at 28.5°C, and staged by morphology as previously described [55]. We used the following mutant and transgenic lines: *Tg(fli1*:*EGFP)* [56], *Tg(sox17*:*GFP)* [15], *Tg(cmlc2*:*EGFP)* [57], *Tg(nkx2*.*5*:*ZsYellow)* [25], *Tg(hsp70*:*dnBmpr1-GFP)* [41], *Tg(hsp70*:*caBmpr1b-GFP)* [58], *swr*^*ta72*^ [44], *Tg(UAS*:*NTR-mCherry)*, *Tg(nkx2*.*3*:*mCherry)*, *Tg(nkx2*.*3*:*EosFP)*, *Tg(nkx2*.*3*:*EGFP-CAAX)* and *Tg(nkx2*.*3*:*KalTA4-p2a-mCherry)*. To generate the *Tg(nkx2*.*3*:*EosFP)*, *Tg(nkx2*.*3*:*EGFP-CAAX)*, and *Tg(nkx2*.*3*:*KalTA4-p2a-mCherry)* transgenic lines, the 5.5 kb of the *nkx2*.*3* upstream regulatory sequence was cloned into a Tol2 transposon-based vector [21].

### Morpholinos and microinjections

Morpholinos were purchased from Gene Tools (Philomath, OR, USA). The standard control morpholino (5’-CCTCTTACCTCAGTTACAATTTATA-3’), *sox32* MO (5’-CAGGGAGCATCCGGTCGAGATACAT-3’), *bmp2b* MO (5’-CGCGGACCACGGCGACCATGATC-3’), *bmp4* MO (5’-GTCTCGACAGAAAATAAAGCATGGG-3’) and *bmp7a* MO (5’-GCACTGGAAACATTTTTAGAGTCAT-3’) were used as previously described [3,59–61]. Microinjections were performed as previously described [62–64].

### Whole-mount *in situ* hybridization and frozen sectioning

Digoxigenin-UTP-labeled RNA probes were synthesized *in vitro* from linearized plasmids using the MEGAscript Kit (Ambion) according to the manufacturer’s instructions. Whole-mount *in situ* hybridizations and frozen sections were performed according to previously published methods [62–64]. For double fluorescence *in situ* hybridizations, anti-digoxigenin-POD (1:400; 11633716001, Roche) and anti-fluorescein-POD (1:400; 11426346910, Roche) were used as primary antibodies to detect digoxigenin-labled *pdgfαa* probes and fluorescein-labeled *nkx2*.*3* probes, respectively. The signals were detected using a TSA system, including Cyanine 3 amplification reagent (FP1170, PerkinElmer), Fluorescein amplification reagent (FP1168, PerkinElmer), and 1x plus amplification diluents (FP1135, PerkinElmer).

### Immunofluorescent staining

Immunofluorescent staining was performed as previously described [3]. Embryos were stained with the following affinity-purified antibodies: anti-GFP (1:1000; A-11120, Invitrogen), anti-Zn8 (1:500; ZDB-ATB-081002-19, Zebrafish International Resource Center), and anti-p-Smad1/5/8 (1:200; 9511, Cell Signaling Technology). Whole-mount embryos were then imaged using a Nikon A1R+ confocal microscope.

### Whole-mount RNAscope assay

Whole-mount RNAscope assay was conducted using the RNAscope Fluorescent Multiplex Reagent Kit (320850, ACD) according to published protocols with some modifications [65]. Without digestion pretreatment, embryos were hybridized using a mixture of *egfp* (400281-C1, ACD) and *bmp2b* RNAscope probes (456471-C2, ACD). For labeling, Amp4 Alt A-FL (320855, ACD) was used. Preamplifier hybridization of Amp1 and amplifier hybridization of Amp3 were both extended to 3 hours. Signal enhancement of Amp2 and labelling with Amp4 were both extended to 1.5 hours. All images were taken using a Nikon A1R+ confocal microscope.

### EosFP photoconversion and lineage tracing

For the EosFP photoconversion and lineage tracing experiments, all embryos were anesthetized and embedded in 0.8% low-melt agarose (0815, Amresco) in glass-bottom dishes (D35-14, Cellvis). EosFP fluorescent proteins were converted upon irradiations with violet-blue light (405 nm) using a Nikon A1R+ confocal microscope (20× objective) until the green fluorescence in the regions of interest disappeared. For lineage tracing, converted embryos were immediately imaged, removed from the agarose, and raised in dark conditions until later evaluation. All confocal stack pictures were processed using Nikon NIS-Elements AR 4.13.00 software.

### Pharmacological treatment and heat shock

Pharmacological treatment was performed by incubating live embryos in the dark in Holtfreter’s solution supplemented with inhibitors as follows: Dorsomorphin (10 μM or 20 μM; P5499, Sigma), DMH1 (10 μM; D8946, Sigma), U0126 (50 μM; U120, Sigma), SP600125 (4 μM; S5567, Sigma), SB203580 (50 μM; S8307, Sigma), CCT036477 (10 μM; SML0151, Sigma), and Metronidazole (MTZ) (50 μM; M3761, Sigma). Control embryos were treated with equivalent amounts of DMSO (0231, Amresco). MTZ was added as early as the 32-cell stage and washed out until 17 somite stage. For heat shock, embryos were subjected to 39.5°C heat shocks for 20 min at 9 hpf and then incubated at 28.5°C until harvested.

## Supporting information

S1 Fig *nkx2.3* is expressed in the pericardium. (**A**) *Tg(nkx2*.*3*:*mCherry;cmlc2*:*EGFP)* embryos at 28 hpf with mCherry fluorescence (red) in the pericardium and EGFP fluorescence (green) in the heart. Single plane images showed that the GFP-positive myocardial cells were surrounded by the mCherry-positive cells. Scale bar, 50 μm. (**B**) *In situ* hybridization of *nkx2*.*3* in whole mount embryos at 36 hpf (left panel, ventral view with anterior to the top). Sagittal section of the same embryo was shown in the right panel.
(TIF)

S2 Fig *nkx2.3*^+^ cells and *nkx2.5*^+^ progenitors belong to different cell types. (**A-D**) mCherry and ZsYellow fuorescence in *Tg(nkx2*.*3*:*mCherry;nkx2*.*5*:*ZsYellow)* embryos at indicated developmental stages. Single plane images showed that no cells within the paired cords (A-C) and the pericardium (D) were double-positive for mCherry and ZsYellow. Dorsal views with anterior to the top. Scale bars, 50 μm.
(TIF)

S3 Fig mCherry-positive cells in the pericardium are not affected in *sox32* morphants. mCherry fluorescence in the pericardium of *Tg(nkx2*.*3*:*mCherry;sox17*:*GFP)* embryos injected ng cMO or ng *sox32* MO at 36 hpf. Ventral views, anterior to the top. Scale bar, 50 μm.
(TIF)

S4 Fig *nkx2.3*^+^ progenitors contribute to the pericardium. (**A**) *Tg(nkx2*.*3*:*EosFP)* embryos were photoconverted in the right-side *nkx2*.*3*^+^ cluster at the 17-somite stage. At 36 hpf, embryos were imaged in the green and red channels in the pericardium. Ventral views, anterior to the top. Scale bar, 50 μm. (**B**) *Tg(nkx2*.*3*:*EosFP)* embryos were photoconverted in the right-side *nkx2*.*3*^+^ cluster at the 10-somite stage, and then these embryos were imaged in the pericardium at 28 hpf. Scale bar, 50 μm.
(TIF)

S5 Fig NTR-mediated ablation of *nkx2.3*^+^ progenitors impairs pericardium development but not endoderm formation. (**A-B**) *Tg(nkx2*.*3*:*KalTA4-p2a-mCherry;UAS*:*NTR-mCherry)* embryos were treated with 50 mM MTZ during different time intervals and then harvested at the indicated developmental stages for *in vivo* confocal imaging (A) and *in situ* hybridization (B). Scale bars, 50 μm. (**C-D**) Depletion of *nkx2*.*3*^+^ progenitors has no obvious effect on endoderm formation. *Tg(nkx2*.*3*:*KalTA4-p2a-mCherry;UAS*:*NTR-mCherry;sox17*:*GFP)* or *Tg(nkx2*.*3*:*KalTA4-p2a-mCherry;UAS*:*NTR-mCherry)* embryos were treated with 50 mM MTZ from the 32-cell stage to the 17-somite stage. Then these embryos were subjected to *in vivo* confocal imaging (C) and *in situ* hybridizations (D) at the 17-somite stage. In panel D, embryos are viewed from the dorsal aspect, and the white dotted lines indicate the region of the pericardium. Scale bars, 50 μm. (**E-F**) Depletion of *nkx2*.*3*^+^ progenitors leads to obvious pericardial edema. *Tg(nkx2*.*3*:*KalTA4-p2a-mCherry;UAS*:*NTR-mCherry)* embryos were treated with 50 mM MTZ from the 32-cell stage to the 17-somite stage, and then these embryos were harvested at 28 hpf for *in vivo* confocal imaging (E, ventral views, anterior to the top; Scale bar, 50 μm). Their morphological defects were shown in (F, lateral views with anterior to the left; Scale bar, 100 μm). Red Arrowheads indicate the pericardium.
(TIF)

S6 Fig Blocking BMP signaling at early somite stages does not affect the development of pan-endoderm. *Tg(sox17*:*GFP)* embryos were treated with 10 μM DMH1 from bud stages until harvested for confocal imaging. Dorsal views with anterior to the top. Scale bars, 50 μm.
(TIF)

S7 Fig Injection of *bmp4* MO and *bmp7a* MO efficiently leads to developmental defects. (**A-B**) Knockdown of *bmp4* perturbed asymmetrical left-right patterning. *Tg(cmlc2*:*EGFP)* embryos was injected with ng *bmp4* MO at one-cell stage. Defects in cardiac jogging was visualized by EGFP expression at 30 hpf. Different types of EGFP expression fluorescence in the heart were shown in ventral views (A). The ratios were shown in (B). Scale bars, 50 μm. (**C-D**) Knockdown of *bmp7a* resulted in a range of dorsalized phenotypes. Wild-type embryos were injected with ng *bmp7a* MO at the one-cell stage and imaged at 36 hpf. Representative dorsalized morphologies (C1-C3) are shown in (C) and their ratios are shown in (D). Scale bar, 100 μm.
(TIF)

S8 Fig Endoderm formation is not affected in *swr^ta72^* mutants. The *sox17* expression in *swr*^*ta72*^ embryos at the bud stage. The *swr*^*ta72*^ mutant embryos can be easily recognized owing to their elongated shape. Note that the *swr*^*ta72*^ mutants showed nearly normal endoderm specification but delayed convergence of endodermal cells.
(TIF)

S9 Fig An integrated model for the specification of pouch progenitors by ectoderm-derived BMP2b. During the early somite stages, the ectodermal cells (orange) express and secret BMP2b proteins (yellow), which play an essential role in the specification of pouch progenitors (pink) from adjacent pharyngeal endoderm (green). PPP, pharyngeal pouch progenitor.
(TIF)
