# Supplementary figures and images for: BMP signaling is required for nkx2.3-positive pharyngeal pouch progenitor specification in zebrafish
Source: PLoS Genet. 2019 Feb 14;15(2):e1007996. doi: 10.1371/journal.pgen.1007996 (PMC6392332; doi:10.1371/journal.pgen.1007996)

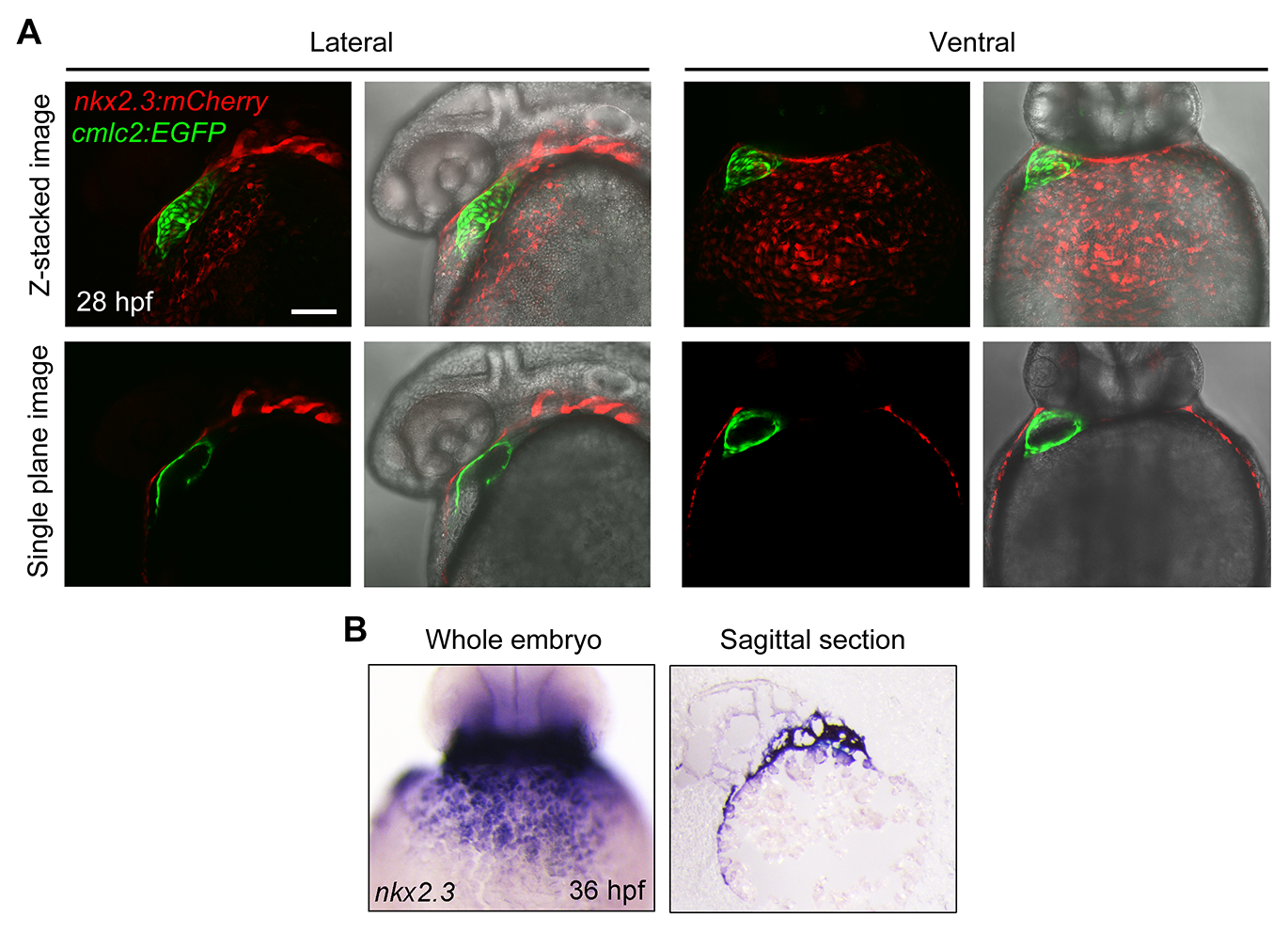

Supplement: S1 Fig — (A) Tg(nkx2.3:mCherry;cmlc2:EGFP) embryos at 28 hpf with mCherry fluorescence (red) in the pericardium and EGFP fluorescence (green) in the heart. Single plane images showed that the GFP-positive myocardial cells were surrounded by the mCherry-positive cells. Scale bar, 50 μm. (B) In situ hybridization of nkx2.3 in whole mount embryos at 36 hpf (left panel, ventral view with anterior to the top). Sagittal section of the same embryo was shown in the right panel. (TIF) [file pgen.1007996.s001.tif]

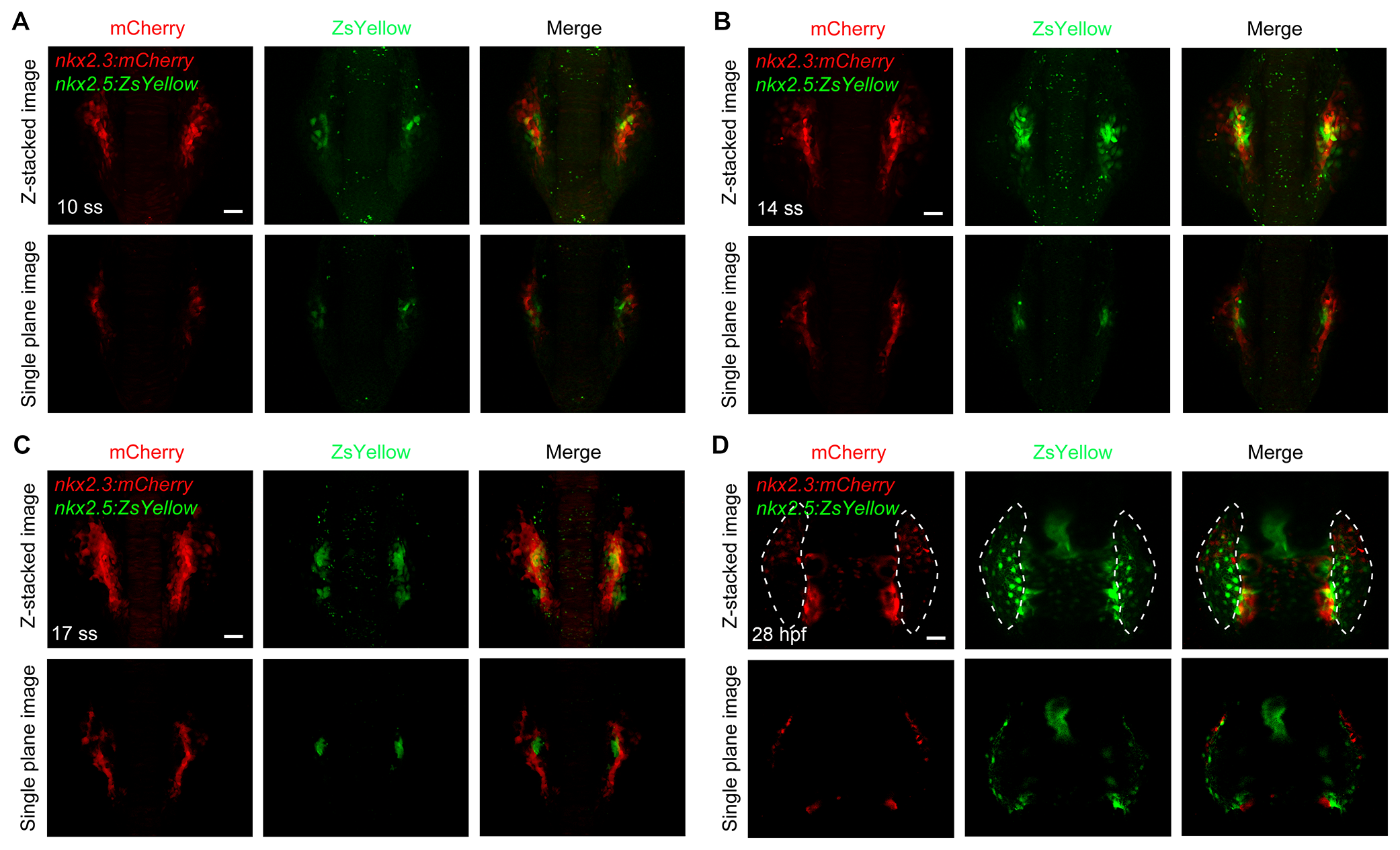

Supplement: S2 Fig — (A-D) mCherry and ZsYellow fuorescence in Tg(nkx2.3:mCherry;nkx2.5:ZsYellow) embryos at indicated developmental stages. Single plane images showed that no cells within the paired cords (A-C) and the pericardium (D) were double-positive for mCherry and ZsYellow. Dorsal views with anterior to the top. Scale bars, 50 μm. (TIF) [file pgen.1007996.s002.tif]

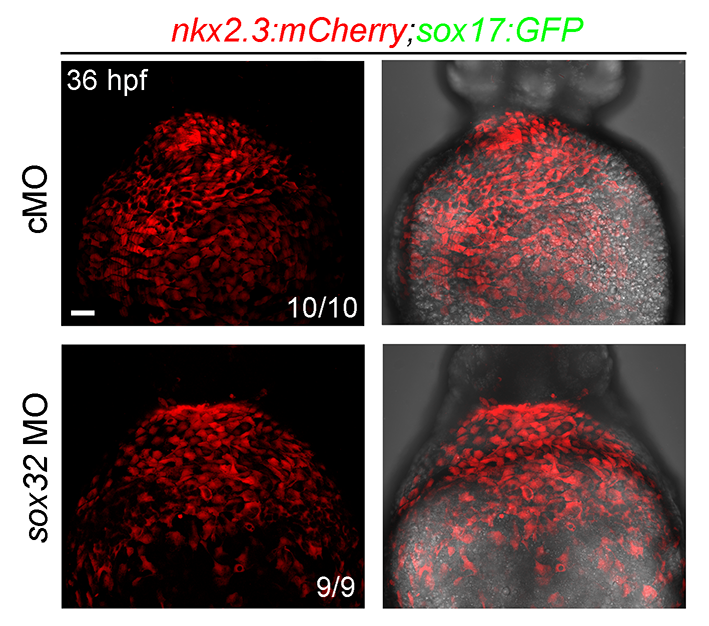

Supplement: S3 Fig — mCherry fluorescence in the pericardium of Tg(nkx2.3:mCherry;sox17:GFP) embryos injected ng cMO or ng sox32 MO at 36 hpf. Ventral views, anterior to the top. Scale bar, 50 μm. (TIF) [file pgen.1007996.s003.tif]

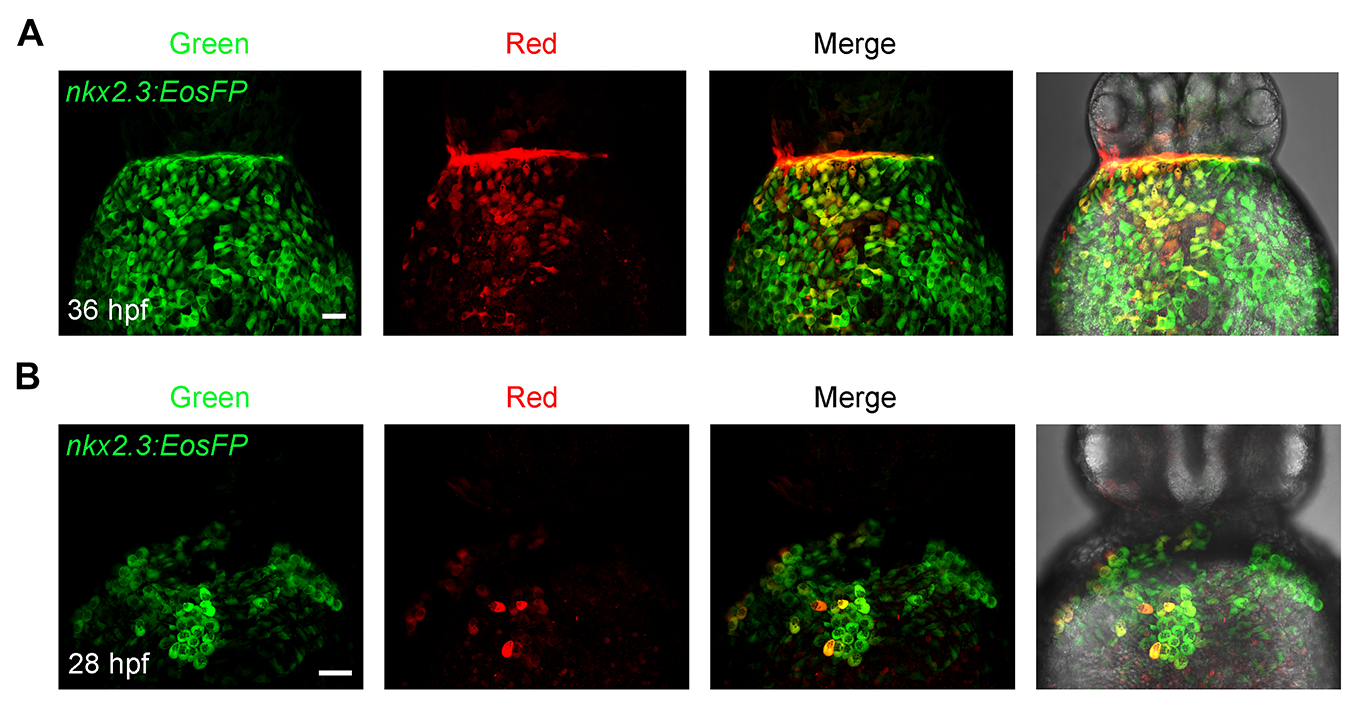

Supplement: S4 Fig — (A) Tg(nkx2.3:EosFP) embryos were photoconverted in the right-side nkx2.3+ cluster at the 17-somite stage. At 36 hpf, embryos were imaged in the green and red channels in the pericardium. Ventral views, anterior to the top. Scale bar, 50 μm. (B) Tg(nkx2.3:EosFP) embryos were photoconverted in the right-side nkx2.3+ cluster at the 10-somite stage, and then these embryos were imaged in the pericardium at 28 hpf. Scale bar, 50 μm. (TIF) [file pgen.1007996.s004.tif]

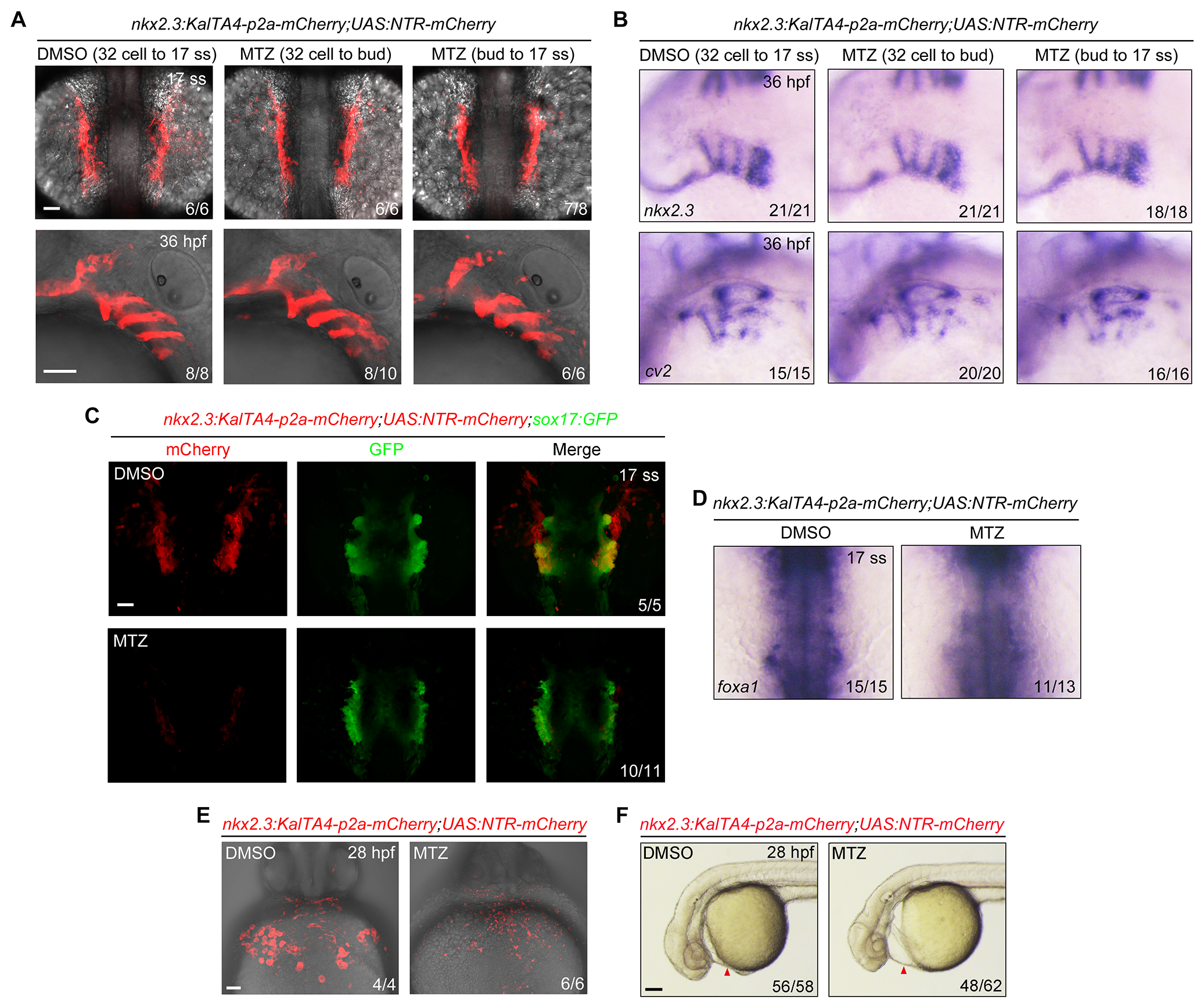

Supplement: S5 Fig — (A-B) Tg(nkx2.3:KalTA4-p2a-mCherry;UAS:NTR-mCherry) embryos were treated with 50 mM MTZ during different time intervals and then harvested at the indicated developmental stages for in vivo confocal imaging (A) and in situ hybridization (B). Scale bars, 50 μm. (C-D) Depletion of nkx2.3+ progenitors has no obvious effect on endoderm formation. Tg(nkx2.3:KalTA4-p2a-mCherry;UAS:NTR-mCherry;sox17:GFP) or Tg(nkx2.3:KalTA4-p2a-mCherry;UAS:NTR-mCherry) embryos were treated with 50 mM MTZ from the 32-cell stage to the 17-somite stage. Then these embryos were subjected to in vivo confocal imaging (C) and in situ hybridizations (D) at the 17-somite stage. In panel D, embryos are viewed from the dorsal aspect, and the white dotted lines indicate the region of the pericardium. Scale bars, 50 μm. (E-F) Depletion of nkx2.3+ progenitors leads to obvious pericardial edema. Tg(nkx2.3:KalTA4-p2a-mCherry;UAS:NTR-mCherry) embryos were treated with 50 mM MTZ from the 32-cell stage to the 17-somite stage, and then these embryos were harvested at 28 hpf for in vivo confocal imaging (E, ventral views, anterior to the top; Scale bar, 50 μm). Their morphological defects were shown in (F, lateral views with anterior to the left; Scale bar, 100 μm). Red Arrowheads indicate the pericardium. (TIF) [file pgen.1007996.s005.tif]

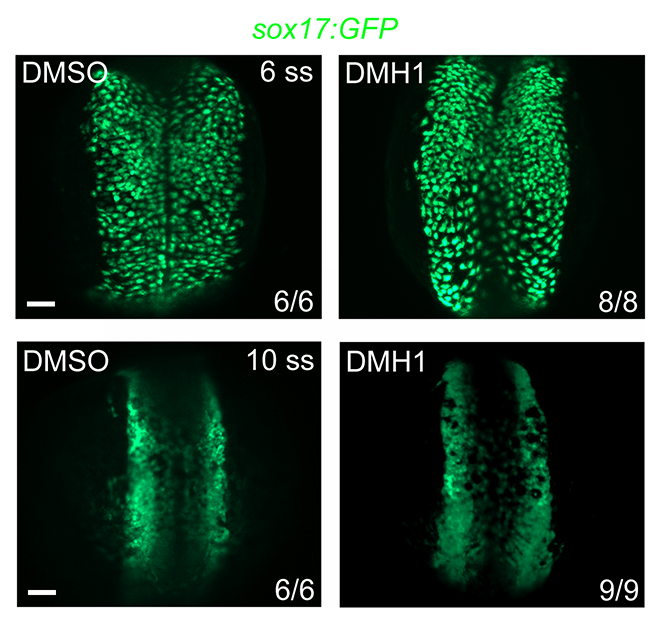

Supplement: S6 Fig — Tg(sox17:GFP) embryos were treated with 10 μM DMH1 from bud stages until harvested for confocal imaging. Dorsal views with anterior to the top. Scale bars, 50 μm. (TIF) [file pgen.1007996.s006.tif]

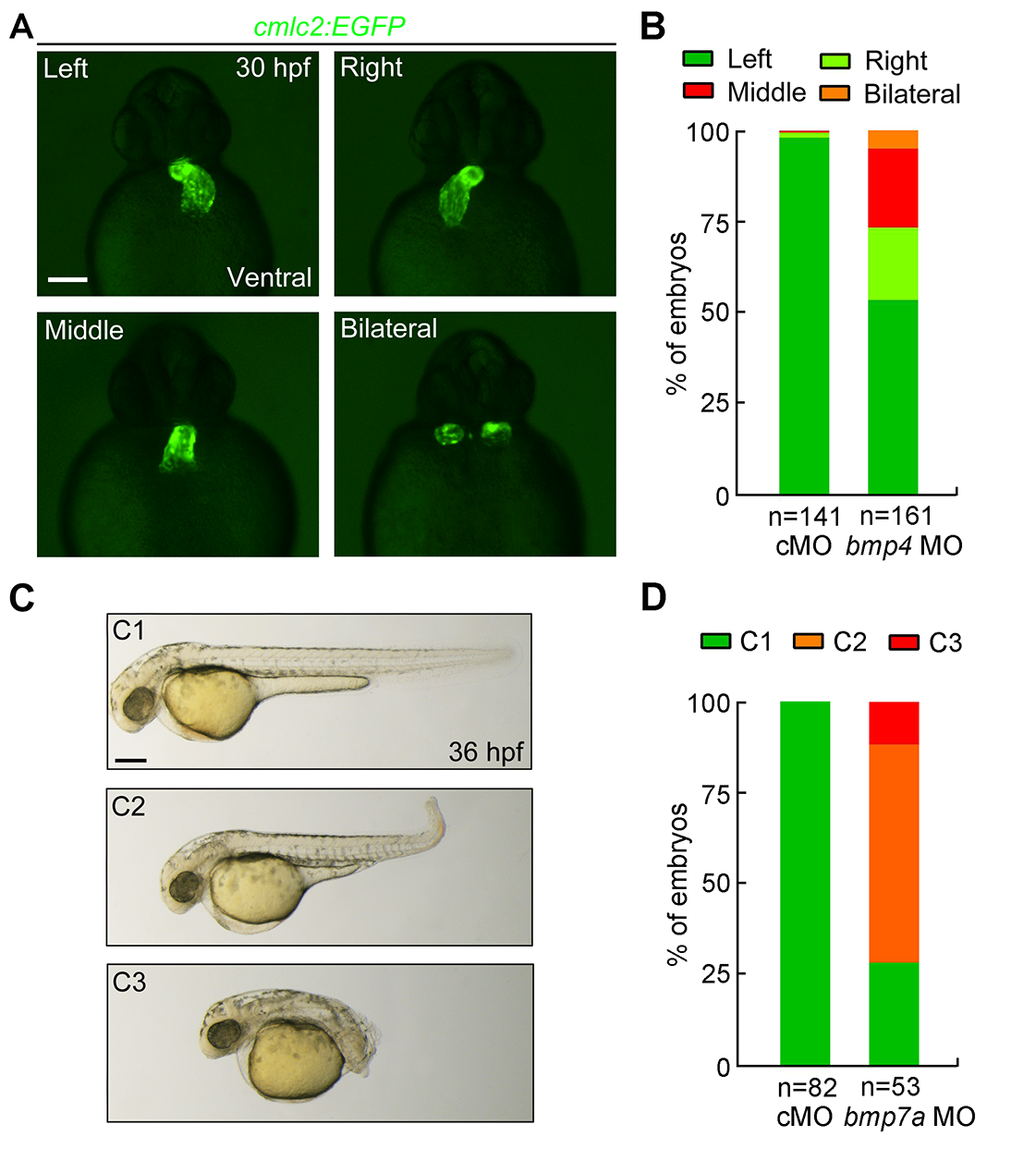

Supplement: S7 Fig — (A-B) Knockdown of bmp4 perturbed asymmetrical left-right patterning. Tg(cmlc2:EGFP) embryos was injected with ng bmp4 MO at one-cell stage. Defects in cardiac jogging was visualized by EGFP expression at 30 hpf. Different types of EGFP expression fluorescence in the heart were shown in ventral views (A). The ratios were shown in (B). Scale bars, 50 μm. (C-D) Knockdown of bmp7a resulted in a range of dorsalized phenotypes. Wild-type embryos were injected with ng bmp7a MO at the one-cell stage and imaged at 36 hpf. Representative dorsalized morphologies (C1-C3) are shown in (C) and their ratios are shown in (D). Scale bar, 100 μm. (TIF) [file pgen.1007996.s007.tif]

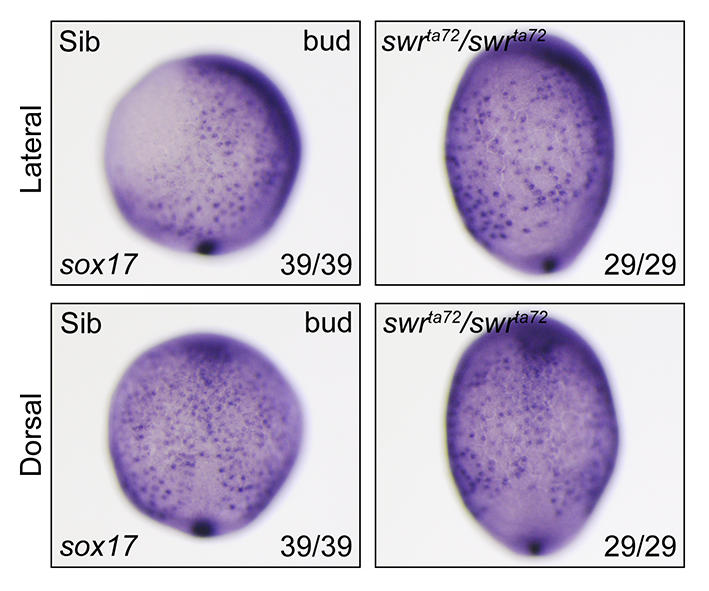

Supplement: S8 Fig — The sox17 expression in swrta72 embryos at the bud stage. The swrta72 mutant embryos can be easily recognized owing to their elongated shape. Note that the swrta72 mutants showed nearly normal endoderm specification but delayed convergence of endodermal cells. (TIF) [file pgen.1007996.s008.tif]

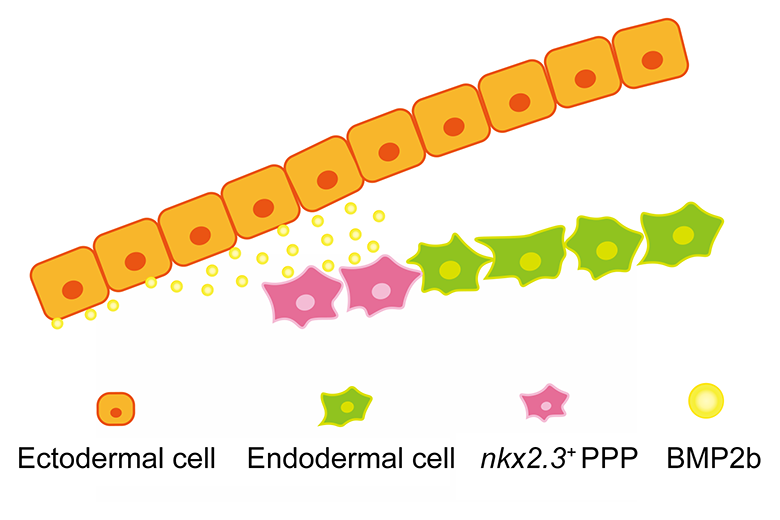

Supplement: S9 Fig — During the early somite stages, the ectodermal cells (orange) express and secret BMP2b proteins (yellow), which play an essential role in the specification of pouch progenitors (pink) from adjacent pharyngeal endoderm (green). PPP, pharyngeal pouch progenitor. (TIF) [file pgen.1007996.s009.tif]
